# Interventions for reducing violence against children in low‐ and middle‐income countries: An evidence and gap map

**DOI:** 10.1002/cl2.1120

**Published:** 2020-10-20

**Authors:** Prachi Pundir, Ashrita Saran, Howard White, Ramya Subrahmanian, Jill Adona

**Affiliations:** ^1^ Campbell Collaboration Vasant Kunj Delhi India; ^2^ Campbell South Asia Vasant Kunj Delhi India; ^3^ UNICEF Office of Research—Innocenti Florence Italy; ^4^ Asian Development Bank Manila Philippines

## Abstract

**Background:**

More than half of the children in the world experience some form of interpersonal violence every year. As compared with high‐income countries, policy responses in low‐ and middle‐income countries (LMICs) are limited due to resource constraints and paucity of evidence for effective interventions to reduce violence against children in their own contexts, amongst other factors.

**Objectives:**

The aim of this evidence and gap map (EGM) is to provide an overview of the existing evidence available and to identify gaps in the evidence base on the effectiveness of interventions to reduce violence against children in LMICs. This report covers evidence published in English; a follow‐up study is under preparation focusing on evidence in five additional languages—Arabic, Chinese, French, Portuguese and Spanish.

**Methods:**

The intervention‐outcome framework for this EGM is based on INSPIRE—Seven Strategies for Ending Violence against Children, published by WHO and other partners in 2016. The seven strategies include implementation and enforcement of laws; norms and values, safe environment; parent–child and caregiver support; income and economic strengthening; response and support services; education and life skills. The search included both academic and grey literature available online. We included impact evaluations and systematic reviews that assessed the effectiveness of interventions to reduce interpersonal violence against children (0–18 years) in LMICs (World Bank, 2018b). Interventions targeting subpopulation of parents, teachers and caregivers of 0–18 years’ age group were also included. A critical appraisal of all included studies was carried out using standardised tools.

**Results:**

The map includes 152 studies published in English of which 55 are systematic reviews and 97 are impact evaluations. Most studies in the map are from Sub‐Saharan Africa. Education and life skills are the most widely populated intervention area of the map followed by income and economic strengthening interventions. Very few studies measure impact on economic and social outcomes, and few conduct cost‐analysis.

**Conclusion:**

More studies focusing on low‐income and fragile and conflict‐affected settings (FCS) and studying and reporting on cost‐analysis are required to address gaps in the evidence. Most interventions covered in the literature focused on addressing a wide range of forms of violence and harm, which limited understanding of how and for whom the interventions work in a given context, for specific forms of violence. More impact evaluation studies are required that assess specific forms of violence, gendered effects of interventions and on diverse social groups in a given context, utilising mixed methods.

**Abbreviations**
3ie: International Initiative for Impact Evaluation
AIDS: acquired immunodeficiency syndrome
AMSTAR 2: Assessing the Methodological Confidence of Systematic Reviews (a measurement tool)
CRC: United Nations Convention on the Rights of the Child
DID: difference in difference
EGM: evidence and gap map
GDP: gross domestic product
GSHS: Global School‐based Student Health Survey
HIC: High income countries
HIV: human immunodeficiency virus
ILO: International Labour Organisation
INSPIRE: A framework containing seven strategies for ending violence against children (Implementation and enforcement of laws; Norms and values, Safe environments; Parent and Caregiver support; Income and economic strengthening; Response and support services; Education and life skills)
ITT: intention to treat
IV: instrumental variables
LMICs: low‐ and middle‐income countries
PSM: propensity score matching
RCT: randomised controlled trial
RDD: regression discontinuity design
SDG: sustainable development goals
UBA: uncontrolled before after
UN: United Nations
UNDP: United Nations Development Program
UNICEF: United Nations Children's Fund
VAC: violence against children
WASH: water, sanitation and hygiene
WHO: World Health Organization

## 1 PLAIN LANGUAGE SUMMARY

### 1.1 The extent of evidence on interventions to prevent violence against children (VAC) is unevenly distributed both geographically and by intervention

This Campbell‐UNICEF evidence and gap map (EGM) includes interventions and outcomes, showing areas of evidence concentration that can be used to prepare evidence summaries to inform policy decisions, as well as identifying gaps in the evidence base which might benefit from a systematic review, research synthesis or additional impact evaluations.

### 1.2 What is this EGM about?

The EGM provides a visual and interactive display of completed and ongoing studies structured around interventions and outcomes mapped in the INSPIRE framework. The framework includes these seven strategies:

- Implementation and enforcement of laws
- Norms and values
- Safe environments
- Parent and caregiver support
- Income and economic strengthening
- Response and support services
- Education and life skills

The population is children aged 0–18 years; parents, carers and other family members of children aged 0–18 years; and professionals involved in delivering support and services to children aged 0–18 years.

This EGM includes studies on all types of VAC, that is, physical violence, sexual violence and emotional violence.

The report presents findings on interventions addressing specific forms of violence, including corporal punishment, peer violence including bullying, and intimate partner violence.

### 1.3 What studies are included?

The map includes 152 studies: 55 systematic reviews and 97 impact evaluations.

### 1.4 What are the main findings of this map?

Of the included impact evaluations:

- Interventions: Education and life skills (40) is the most commonly studied intervention area to reduce VAC, followed by income and economic strengthening (38) and parent and child caregiver support (27).
- Outcomes: Direct impact on VAC (91) is the most frequently measured outcome, followed by impact on changing norms and values (47) and safety and risk factors for other harms (45). Few impact evaluation studies measured economic and social outcomes (19) and there is a lack of studies conducting cost‐analysis (2).
- Geographical distribution: Most studies are concentrated in Sub‐Saharan Africa (59) and South Asia (13). The concentration of impact evaluations is particularly low for conflict‐affected settings.
- Study confidence: A large number of impact evaluations have methodological limitations and are assessed to be of low confidence (47).

The systematic review evidence base is large, but similar to impact evaluations, is unevenly distributed across regions, with most reviews covering studies in Sub‐Saharan Africa (37) followed by South Asia (27) and East Asia and the Pacific (28).

Of the included systematic reviews, parent, child and caregiver support (21), norms and values (20), and response and support services (20) were the most commonly studied interventions.

Many of the systematic reviews were assessed to have methodological limitations. There are large numbers of reviews rated as being of low and medium confidence, particularly those related to parent, child and caregiver support and to norms and values interventions.

### 1.5 What do the findings of the map mean?

Impact evaluations and systematic reviews of interventions for reducing VAC have increased over the years, but there remain limitations that need to be addressed in research investment priorities and future studies. The evidence base is concentrated in Sub‐Saharan Africa and South Asia, and select countries within these regions. South Africa, Ethiopia and India are the most represented countries.

There should be more studies from low‐income and conflict‐affected settings, and more cost‐analysis studies. Studies focusing on interventions linked to specific forms of violence, rather than multiple or unspecified forms of violence, could strengthen understanding of intervention effectiveness.

Overall, the EGM findings suggest the need to ensure increased investment in research to assess effectiveness of interventions, with specific attention to addressing thematic and geographical gaps.

### 1.6 How up‐to‐date is this EGM?

The authors searched for studies published up to December 2019.

## 2 EXECUTIVE SUMMARY

### 2.1 Background

More than 1 billion children—over half the children in the world—report having experienced some form of violence in a previous year (Hillis, Mercy, Amobi, & Kress, 2016). VAC includes all forms of violence experienced by children aged 18 years and below, whether perpetrated by parents or other caregivers, peers, romantic partners, or strangers (WHO, 2018). As defined by UNICEF, the scope of violence includes, “all forms of physical or mental violence, injury or abuse, neglect or negligent treatment, maltreatment or exploitation, including sexual abuse” (article 19, paragraph 1, of the Convention 1989)

In recognition of the enormity of the scale and impact of childhood violence, global actors have stepped up their focus to advocate for greater investment in violence prevention and response. A technical package supporting seven evidence‐based strategies to end VAC—INSPIRE—developed by the WHO, UNICEF and eight other international agencies and initiatives, has been widely promoted and adopted as an essential tool in supporting national investments and actions towards realising this commitment (WHO, 2016a, 2016b). The Global Partnership to End Violence against Children, also launched in 2016, serves as a global platform aimed at “ending violence against children in every country, every community and every family” (Know Violence in Childhood, 2017) and advocates broadly for the use of INSPIRE to accelerate the implementation of violence prevention interventions. These developments, along with significant global commitments articulated in the sustainable development goals (SDGs), have provided greater impetus for global, regional and national level actions to end violence.

Although considerable research on VAC in high‐income countries (HICs) is available, the same is not true for low‐ and middle‐income countries (LMICs) as defined by World Bank country classification (World Bank, 2018b). The mapping of available evidence and especially the evidence required on effectiveness of interventions to reduce VAC is a priority area for policy and practice in LMICs (UNICEF, 2018b). This report summarises findings of phase 1 from an EGM of evidence published in English commissioned by UNICEF Office of Research—Innocenti and undertaken by the Campbell Collaboration. Phase 2 will include evidence available in five languages; Arabic, Chinese, French, Portuguese and Spanish.

#### 2.1.1 Purpose

The purpose of the EGM is to provide an overview of the state of evidence, inform policy and programming and identify important gaps in evidence where the evidence base might benefit from systematic reviews, another type of research synthesis or new impact evaluation. The construction of an EGM is the first step towards building an evidence architecture to end VAC (White, 2019). The EGM will contribute to broadening the included interventions and outcomes in the INSPIRE framework to better reflect the state of evidence on VAC, and independently provide an updated overview of knowledge and evidence gaps for the field.

#### 2.1.2 Objective

The objective of this map is to provide an overview of the existing evidence base and gaps in evidence aimed at reducing VAC in LMICs using an intervention‐outcome framework based on the INSPIRE framework—developed by the WHO, UNICEF and eight other international agencies and initiatives (WHO, 2016a, 2016b).

The INSPIRE framework lists seven evidence‐based strategies to end VAC:

- Implementation and enforcement of laws
- Norms and values
- Safe environments
- Parent and caregiver support
- Income and economic strengthening
- Response and support services
- Education and life skills

Utilising this framework to code intervention categories, the EGM was developed to:

i. Identify existing gaps in evidence to better inform future investment in research.
ii. Identify clusters of impact evaluations that offer opportunities for evidence synthesis.
iii. Identify, appraise and provide short summaries of existing evidence of the included systematic reviews and impact evaluations.

### 2.2 What is an EGM?

An EGM is a presentation of the available, relevant evidence for a particular sector. It provides a visual display of completed and ongoing systematic reviews and impact evaluation structured around a framework. In the present map, the rows are intervention categories and the columns are indicator (outcome) categories. The present EGM provides an overview of all available evidence in English on the key outcome categories and interventions aimed at reducing VAC in LMICs. The EGM will be updated with evidence from other languages over 2020–2021.

#### 2.2.1 Scope

The scope of our EGM is defined by a framework of intervention and outcome categories and subcategories. We included impact evaluations and systematic reviews of effectiveness studies to reduce VAC. Interventions were categorised by the seven INSPIRE categories, and assessed across seven outcome categories that they reported—(i) direct impact on violence, (ii) norms and values, (iii) economic and social factors, (iv) safety and risk factors for other harms, (v) health, (vi) education outcomes. Additionally, cost‐analysis is included as a seventh outcome category.

On the outcomes, we included studies that reported any of the above outcome categories, provided the intervention is intended to reduce VAC and its risk factors.

#### 2.2.2 Interventions

The search included interventions aimed at addressing different types of violence including physical, sexual and emotional violence. As there are different types and forms of VAC, the report and maps are structured to present findings of interventions that specifically address corporal punishment, intimate partner violence, and peer violence, as the three most common forms of violence for which we found interventions were specifically tailored.

In addition, where interventions do not specify a type or form of violence, where they report more than one form of violence, where they cover broader harms experienced by children, or where interventions include attention to the consequences of exposure to violence, we have categorised them in an “unclassified” group.

Boxes 1 and 2 present the key working definitions that we use throughout the report.

Box 1 **Key concept and definitions used in the EGM**

**Table:** 

Violence against children: Violence against children includes all forms of violence against people under 18 years old, whether perpetrated by parents or other caregivers, peers, romantic partners, or strangers (WHO, 2018).	
***What did this EGM include*?**	
1. The EGM included studies on the effectiveness of interventions on both perpetration of violence against children as well as children victimized by violence.	
2. The EGM included interventions aimed at addressing different types of violence studied included including physical, sexual and emotional violence. As there are different types and forms of violence against children, the report and maps are structured to reflect present findings of interventions that specifically addressed corporal punishment, intimate partner violence, and peer violence, as these were the most common forms of violence for which we found interventions were specifically tailored.	
3. In addition, where interventions do not specify a type or form of violence, where they reportede more than one form of violence, where they covered broader harms experienced by children, or where interventions included attention to the consequences of exposure to violence, we have categorised them in an “unclassified” group.	
***Forms of violence included in the EGM***:	
1. Peer violence/Bullying (including cyber‐bullying)	This is unwanted aggressive behaviour by another child or group of children who are neither sibling nor in a romantic relationship with the victim. It involves repeated physical, psychological or social harm, and often takes place in schools and other settings where children gather, and online (WHO, 2018).
2. Corporal Punishment:	Any punishment in which physical force is used and intended to cause some degree of pain or discomfort, however light. Most involves hitting (“smacking”, “slapping”, “spanking”) children, with the hand or with an implement—a whip, stick, belt, shoe, wooden spoon, etc. But it can also involve, for example, kicking, shaking or throwing children, scratching, pinching, biting, pulling hair or boxing ears, forcing children to stay in uncomfortable positions, burning, scalding or forced ingestion (for example, washing children's mouths out with soap or forcing them to swallow hot spices). In addition, there are other non‐physical forms of punishment that are also cruel and degrading and thus incompatible with the UN Convention on the Rights of the Child. These include, for example, punishment which belittles, humiliates, denigrates, scapegoats, threatens, scares or ridicules the child (UNCRC Committee, 2006).
3. Intimate partner violence (IPV)	The World Health Organisation defines Intimate partner violence as “behaviour by an intimate partner or ex‐partner that causes physical, sexual or psychological harm, including physical aggression, sexual coercion, psychological abuse and controlling behaviours” (WHO, 2014).
4. “Unclassified”	Here we include studies where interventions do not specify a type or form of violence, where they report more than one form of violence, where they cover broader harms experienced by children, or where interventions include attention to the consequences of exposure to violence, we have categorised them in an “unclassified” group.

Box 2 **Definition for outcome categories**

**Table:** 

Violence	Violence including physical, sexual, emotional violence, peer violence/bullying, corporal punishment and intimate partner violence
Norms and values	Any indicators measuring changes in norms, values or beliefs that lead to reduced acceptance to violence against children, favourable attitudes towards gender equity
Health	Any indicators measuring changes related to mental, physical health status, related injuries, mortality, and substance abuse, sexual and reproductive health.
Safety, and risk factors for other harms	Any indicators measuring changes in both public safety as well as individual or community risk factors associated with early marriage, FGM and child labour or incidence of these forms of violence?
Economic and social	Any measures related to impact on social discrimination and social inclusion. These also include impact on related “drivers” of violence against children, i.e., poverty and food security.
Education	These include measures of factors relating to school environment e.g. gender roles and life skills, teacher engagement. These also include measures of factors affecting school performance, as well as overall school performance reports including truancy/exclusion levels, school enrolment.
Cost‐ analysis	These outcome measures the economic cost of violence prevention interventions and highlights economic impact of implementations.

#### 2.2.3 Outcomes

Included studies were those that reported interventions to reduce different types and forms of VAC. Studies were categorised by seven outcome categories on which they reported: direct impact on violence, norms and values, economic and social factors, safety and risk factors for other harms, health and education outcomes (Box 2). Additionally, the report includes cost‐analysis as a seventh outcome category.

### 2.3 Search method

The EGM search method was developed to scan literature in two phases:

**Phase 1 (2019–2020)**: We validated the screening and coding tool through pilot screening and coding from the studies included in the INSPIRE Seven Strategies for Ending Violence against Children (WHO, 2016b).

The next step included an online search of relevant systematic reviews and impact evaluation from academic and grey literature searches from the year January 2000–December 2019.

a. Academic search: An experienced systematic reviewer drafted the search strategy, validated by an experienced information specialist. The search strategy consisted of range of search concepts and associated terms. The search strategy was applied to systematic review databases, such as 3ie and Campbell Collaboration databases; academic databases, such as OVID and Embase (Table 1).
  In total, 2,862 records were identified from the search of academic databases.
b. Limited grey literature search: This included studies from non‐academic sources such as:
  ➣ Organisational websites: such as UNICEF, DFID, WHO, USAID, Action Aid, etc. (Table 1).

**Table 1:** Databases searched

Source	Specific database
Systematic review databases	3ie Systematic Review Database Campbell Collaboration Cochrane Collaboration Collaboration for Environmental Evidence EPPI Centre Evaluation Database of Education Research PROSPERO Research for Development Swedish Agency for Health Technology Assessment and Assessment of Social Services Epistomonikos
Academic databases	Web of Science SCOPUS PubMed ERIC EBSCO Medline PsychInfo EMBASE CABI's Global Health CINAHL EBSCOhost Econlit Eldis Google Scholar IDEAS‐Repec Popline PubMed SciELO Social Science Research Network (SSRN) Sociological abstracts (ProQuest)
Organisational website	DFID (including Research for Development (R4D) ILO IOM Save the Children UN Women UNDP UNFPA Evaluation Database UNHCR UNICEF UNICRI UNODC USAID WHO/PAHO
Other Grey literature sources	Abdul Latif Jameel Poverty Action Lab (J‐PAL) Action against Hunger African Development Bank Association for the Development of Africa CAF Development Bank of Latin America CARE CIFF Child Fund International Gates Foundation GreyNet International Innovations for Poverty Action (IPA) Database International Center for Research on Women International Food Policy Research Institute (IFPRI) International Rescue Committee (IRC) International Red Cross IPC‐IG (Working papers) Joanna Briggs Institute Evidence‐Based Practice Database London School of Hygiene and Tropical Medicine Oak Foundation Opengrey Overseas Development Institute Proquest Dissertations & Theses Sexual Violence Research Initiative (SVRI) Social Care Online UN Economic and Social Council UNESCO UNICEF Innocenti Research Centre Working Group on Early Childhood Development World Bank Group (WBG) World Food Programme World for World Organization World Vision

A total of 185 additional studies were identified from the grey literature search for Title and abstract screening.

Number of included studies after screening for Title and abstract and full text were 152. Reference checking was done for these 152 studies.

Expert consultation: An expert advisory group was established and consulted at every stage of the EGM development, from coding development to analysis of findings including search resources and studies.

**Phase 2** is underway and extends the searches to Arabic, Chinese, French, Portuguese and Spanish.

### 2.4 Selection criteria

The primary population of interest for this map is children and adolescents in the age group of less than or equal to 18 years from LMICs (World Bank, 2018b).

Studies, with multiple populations were included in the map if they include a portion of children under 18 years, had an intervention to reduce VAC and/or its risk factors such as poverty, child marriage, child labour and the study conducted in LMICs.

### 2.5 Screening, data extraction and confidence appraisal

The screening was conducted using the eligibility criteria defined in Supporting Information Appendix 3.

Two independent reviewers screened titles and abstracts and a third reviewer resolved any discrepancies. Two independent reviewers coded the included studies from full text. The coded information includes bibliographic details for the study, the interventions and outcomes from the framework that the study measured and other relevant aspects such as population, region and countries.

Two independent reviewers appraised the confidence of all included studies using standardised tools for systematic reviews, AMSTAR 2 (Shea et al., 2017) and impact evaluations (Modified risk of bias tool). Studies were confidence rated as high, medium or low confidence based on critical appraisal tool findings.

We did NOT exclude any study based on study confidence.

### 2.6 Results

#### 2.6.1 Total number of studies and their type

The map includes 152 studies of which 55 are systematic reviews and 97 are impact evaluations. The impact evaluations predominantly include randomised controlled trials (RCTs)—51 of the impact evaluation are RCTs. The other study designs were quasi‐experimental‐ before and after design (35), matching/propensity score matching methods (4), difference in difference (4) and regression discontinuity design (3).

#### 2.6.2 Distribution of impact evaluations across intervention and outcome categories of INSPIRE

The studies are unevenly distributed across intervention and outcome areas. Education and life skills is the most widely populated area of the map. Nearly 43 out of 97 impact evaluations have interventions related to education and life skills. Income and economic strengthening is the next most populated area with 37 studies followed by parent, child and caregiver support with 29 studies, and norms and value with 23 studies. The most common outcome measure is direct impact on violence (n = 92), followed by norms and values (46) and safety and risk factors (45). Very few studies measured impact on economic and social outcomes (18) and only two studies conducted cost‐analysis.

#### 2.6.3 Distribution of systematic reviews across intervention and outcome categories of INSPIRE

Parent‐child and caregiver support and norms and values are most widely populated with 21 reviews each, closely followed by education and life skills (20) and response and support services (20) and income and economic strengthening with 15 reviews. Limited evidence was identified on safe environment (4) and laws, crime and justice (2). The most common outcome measure is direct impact on violence (53), followed by norms and values (23), health (19) and safety and risk factors (15). Very few studies measured impact on economic and social outcomes (3).

#### 2.6.4 Confidence in study findings

The systematic reviews and impact evaluations included were assessed for the level of confidence that could be placed in their findings. Only 17% of the 55 systematic reviews included are high‐confidence systematic reviews, which means that more than three quarters (83%) are either low or medium confidence systematic reviews (18 and 25, respectively). A similar picture emerges for impact evaluations, where only 30% of the 97 included impact evaluations were rated as high confidence, which means that 70% were rated as either low of medium confidence (47 and 20, respectively).

#### 2.6.5 Geographic distribution

The EGM provides us with the opportunity to analyse the distribution of studies within regions. For instance, the distribution of impact evaluations is uneven across regions, with a concentration in sub‐Saharan Africa (59), followed by South Asia (13), Latin America and the Caribbean (11), East Asia and Pacific (8), Middle East and North Africa (4), and Europe and Central Asia (3). Within regions, the distribution is further concentrated in a few countries and on a few intervention areas.

#### 2.6.6 Age distribution

Most of the impact evaluation and systematic reviews address adolescent populations of 10‐18 years (111 studies). Studies assessing the impact of interventions on children less than three years old are sparse (15). There are also evidence gaps in studies of children belonging to ethnic minorities or low‐income groups or children with disabilities.

#### 2.6.7 Distribution by forms of violence

We identified 38 studies of interventions addressing intimate partner violence against adolescents by romantic partners, 19 studies addressing interventions for corporal punishment either by parents or caregiver or teachers and 7 studies of interventions addressing peer violence and bullying. Of these 38 studies reporting interventions to address intimate partner violence, 33 reported direct impacts on physical violence, 31 on sexual violence and 28 on emotional violence. Other outcomes reported were norms and values (22), health (12) and education (8). The remaining studies were categorised as “unclassified” where interventions do not specify a type or form of violence, where they report more than one form of violence, where they cover broader harms experienced by children, or where interventions include attention to the consequences of exposure to violence.

#### 2.6.8 Perpetration of violence

We found only a handful of studies assessing the effectiveness of interventions on perpetration of violence. The majority of studies focused on interventions for victims of violence. Thirteen (13) studies focused on parent/caregivers, 11 on romantic partner/intimate partner, 11 on peers, 3 studies on teachers as perpetrators.

### 2.7 Conclusion 

Our study reveals that rigorous evidence synthesis on the interventions for VAC remains limited in many areas. There is concentration of impact evaluations in education and life skills and income and economic strengthening, specifically on safe environment where the systematic reviews are scarce. Also, most of the evidence is found to be concentrated in few countries in Sub‐Saharan Africa and South Asia with South Africa, Ethiopia and India being the major countries of coverage. More studies focusing on low‐income and conflict‐affected settings are needed because violence affecting children is anticipated to be more prevalent in such settings. More impact evaluations are needed to assess the impact of more than one kind of strategy, evaluate effects for vulnerable populations, as well as report on cost‐ analysis of interventions. The focus of majority of studies has been on impact of interventions on victims of violence or their parent/caregivers or teachers; however, there seemed to be gaps in studies assessing the impact of interventions on perpetration of violence. There is a need to conduct high‐confidence impact evaluations and systematic reviews.

## 3 BACKGROUND

### 3.1 Introduction

#### 3.1.1 The problem, condition or issue

VAC includes all forms of violence under 18 years of age, whether perpetrated by parents or other caregivers, peers, romantic partners, or strangers (WHO, 2018). As defined by UNICEF, the scope of violence includes, “all forms of physical or mental violence, injury or abuse, neglect or negligent treatment, maltreatment or exploitation, including sexual abuse” (article 19, paragraph 1, of the Convention, 1989). It includes maltreatment, bullying, youth violence, intimate partner violence, sexual violence and emotional or psychological violence (detailed definition given in Supporting Information Appendix 1).

More than 1 billion children between two to seventeen years—half the children in the world—report having experienced some form of interpersonal violence in a previous year (Hillis et al., 2016) Violence affects children in LMICs as well as in HICs; however, the burden and types of violence vary substantially between economies. Violence, exploitation and abuse against children occur in the homes, families, schools, care and other institutions, justice systems, workplaces and communities across all contexts, including online, and including as a result of conflict and natural disasters (UNICEF, 2011). Globally, three in four children aged two to four worldwide experience violent disciplines by their caregivers on a regular basis; six in ten children are punished by physical means (UNICEF, 2017). Violent discipline also takes place in schools: one in two school‐age children between six and 17 years live in countries where corporal punishment at school is not fully prohibited (UNICEF, 2017). Gender‐based discrimination places girls at high risk of violence and its experience and impacts extend throughout the life course. The use of corporal punishment against adolescent girls is widespread and 84 million adolescent girls aged 15–19 have been victims of emotional, physical or sexual violence at the hands of their husbands or partners at some point in their lives (UNICEF, 2014). A few striking features of childhood violence have drawn substantial international attention in the recent years. First, violence exposure starts early in childhood including through experience of corporal punishment recorded as early as age 1 (Devries et al., 2018; Know Violence in Childhood, 2017). Second, much of the violence experienced by children is at the hands of adults who are typically to be found within a circle of trust and caregiving—parents, teachers, neighbours and authority figures. Third, there is also a striking rise in peer violence as children grow older and violence spills into peer relationships including through bullying (offline and online), dating and intimate partner violence, as well as gang violence (Know Violence in Childhood, 2017). The gendered experience of violence also manifests as children grow older and transition to adolescence.

Vulnerability to violence is also experienced by children who face other violations of their rights such as early marriage or child labour. For instance, girls who marry before 18 are more likely to experience intimate partner violence than their peers who marry later (UNICEF, 2005a).

#### 3.1.2 Risk factors and context

Data suggest that risk factors for exposure to or experience of violence manifest differently across gender, age groups and context. WHO estimates that the highest child homicide rates occur in adolescents, especially boys, aged 15–17 years and among 0–4‐year‐old children (WHO, 2004). A 2011 review estimated the global lifetime prevalence of childhood sexual abuse to be about 18% for girls compared with 8% for boys (Stoltenborgh, Van IJzendoorn, Euser, & Bakerman‐Kranenburg, 2011). Girls all over the world are victims of child marriage, forced pregnancy, and female genital mutilation, particularly in LMICs. Despite wide variation by country, physical punishment is common in LMICs (Lansford & Deater‐Deckard, 2012). Prevailing social norms supporting harsh punishment of children and women and inadequate family support services and parenting practices have been documented in the context of physical punishments (Lansford, Deater‐Deckard, Bornstein, Putnick, & Bradley, 2014).

#### 3.1.3 Impact of VAC

The severity of the effects of violence experienced by children is magnified because of the serious intergenerational impacts on the future wellbeing of children as they transition to adulthood. Experience of violence during childhood increases the risk of becoming victims or perpetrators of violence during adulthood (WHO, 2016b). Violence can negatively affect physical, mental, sexual, and reproductive health, and may increase the risk of acquiring HIV in some settings (WHO, 2017). VAC is associated with poor educational outcomes, economic insecurity including food insecurity, parental unemployment, inadequate housing and other basic necessities for children and families in LMICs (Peterman, Neijhoft, Cook, & Palermo, 2017). The global costs related to physical, psychological and sexual VAC have been estimated to be between 3% and 8% of global GDP (Pereznieto, Montes, Routier, & Langston, 2014).

#### 3.1.4 Global attention to ending violence through prevention

Although experiencing violence in childhood impacts lifelong health and wellbeing, such violence is often preventable (WHO, 2018). The SDG provide a comprehensive framework within which the UN General Assembly has made a global commitment to ending all forms of VAC (Box 3), with a specific target [SDG 16.2] was included in the 2030 Agenda for Sustainable Development giving renewed impetus towards the realisation of the right of every child to live free from fear, neglect, abuse and exploitation.

Box 3 **SDG agenda 2030 targets to end violence against children**

**Table:** 

The SDG 2030 Agenda includes a specific target to end all forms of violence against children (16.2). Abuse, neglect and exploitation of children are also mainstreamed across several other targets. The following goals and targets are particularly relevant for ending violence against children:
Goal 5: Achieve gender confidence and empower all women and girls
5.2 Eliminate all forms of violence against all women and girls in public and in private spheres, including trafficking and sexual and other types of exploitation. 5.3 Eliminate all harmful practices, such as child, early and forced marriage, and female genital mutilation
Goal 8: Promote sustained, inclusive and sustainable economic growth, full and productive employment and decent work for all
8.7 Take immediate and effective measures to eradicate forced labour, end modern slavery and human trafficking and secure the prohibition and elimination of the worst forms of child labour, including recruitment and use of child soldiers, and by 2025 end child labour in all forms.
Goal 16: Promote peaceful and inclusive societies for sustainable development, provide access to justice for all and build effective, accountable and inclusive institutions at all levels
16.1 Significantly reduce all forms of violence and related death rates everywhere.
16.2 End abuse, exploitation, trafficking and all forms of violence against and torture of children.

Several SDG targets address specific forms of violence such as eliminating violence against women and girls in public and private spheres [SDG 5.2], eliminating harmful practices towards children such as child marriage and female genital mutilation [target 5.3], and the eradication of child labour, including the recruitment and use of child soldiers [target 8.7]. The year 2019 also marked the 30th anniversary of the adoption of the UN Convention on the Rights of the Child (CRC), which provides an important opportunity to galvanise further attention to the importance of ending VAC.

The Global Partnership to End Violence against Children, launched in 2016, serves as a global platform with the aim of “ending violence against children in every country, every community and every family” (End Violence against children, 2018). The Global Partnership has 30 “path finding” countries committed to investing in the technical package INSPIRE that promotes seven evidence‐based strategies to end VAC, developed by the WHO and nine other international agencies and initiatives. INSPIRE has been widely promoted and adopted as an essential tool in supporting national investments and actions towards realising this commitment (WHO, 2016b). Global evidence synthesised in reports such as *Ending Violence in Childhood: Global Report* 2017 makes a strong case for the prevention of childhood violence and expanding investments in evidence‐informed strategies. Collectively all these initiatives, resources and platforms have created a space to develop a stronger evidence architecture to inform the acceleration of local, national, regional and global actions to end VAC.

#### 3.1.5 The EGM

##### What does this EGM include?

This EGM focuses specifically on violence experienced by children whether perpetrated by adults or peers.

The EGM is focused on effectiveness studies of interventions where the primary aim is the reduction of VAC.

##### Approach

The INSPIRE framework has been identified as the basis for the intervention‐outcome framework for the present EGM (WHO, 2016a, 2016b). In 2018, follow‐up resources on INSPIRE technical package were published. “INSPIRE indicator guidance and results framework” and “INSPIRE handbook: action for implementing the seven strategies” were the two resources to improve the implementation and use of the guidelines (WHO, 2018). The “INSPIRE handbook” explains in detail how to choose and contextually implement interventions and “Indicator Guidance and Results Framework” is designed to help governments and agencies to monitor progress and track change over time when INSPIRE strategies are implemented.

The INSPIRE framework supports efforts to achieve the SDG by aiming to reduce significantly all forms of violence and related death rates everywhere.

The intervention‐outcome framework used in the EGM is based on the INSPIRE framework (WHO, 2016b) which identifies seven evidence‐based strategies to prevent VAC:

1. Implementation and enforcement of laws;
2. Norms and values;
3. Safe environments;
4. Parent and caregiver support;
5. Income and economic strengthening;
6. Response and support services; and
7. Education and life skills

The intervention categories are based on the seven strategies of INSPIRE; in addition, subcategories have been developed based on pilot coding of 30 key studies, expert advice and discussion within the team and advisory members. These are detailed in Chapter 4 of the report as well as in Supporting Information Appendix 1.

We use an adapted category for the first pillar of INSPIRE implementation and enforcement of laws, which we have expanded to include laws, crime, and justice in the present map. This deviation was based on the pilot coding of studies.

##### What does this EGM aim to achieve?

To support global efforts to end violence, there is an increased need to invest in generating sound evidence on effective strategies to prevent or end VAC, in order to inform national and global actions as well as to point to the evidence gaps that need attention, and to those areas where evidence can be better utilised to inform policies and investments. The EGM aims to achieve this by collating available evidence and identifying gaps in evidence base where research might benefit from a systematic review, research synthesis or a impact evaluations.

In order to move towards the ambitious targets laid out in the SDG, it is likely that substantial improvements in resource allocation are needed to promote interventions which are effective in improving outcomes in particular contexts. The purpose of this EGM is to assist policy makers, researchers and practitioners in gaining access to available evidence on the effectiveness of interventions to reduce VAC. Mapping the distribution and focus of available evidence will help both identifying areas where new impact evaluations are required, and evidence synthesis gaps where studies exist but require consolidation to understand findings. The EGM does not replace other impact evaluations or address specific methodological or other gaps, but instead lays out an assessment of evidence availability in order to guide further research investment.

##### Rationale

Collecting reliable data on violence poses a challenge both in terms of gathering sensitive information as well as gaining access to vulnerable children in an ethical and safe way (UNICEF, 2005b). Governments in many HICs have recognised the high social and economic costs imposed by violence and introduced contextual interventions for preventing and responding to VAC. In many LMICs this is as yet not necessarily the case, and interventions are often the result of external funding and technical assistance rather than government endorsed initiatives. The SDGs’ articulation of the global commitment to end violence against women and children, platforms such as the Global Partnership to End Violence against Children, and the active role played by many national organisations and movements all play an important role in increasing national governments’ attention to the issue.

#### 3.1.6 Existing EGMs and/or relevant systematic reviews

The Child Wellbeing Mega Map (UNICEF, 2018b) was a basis for developing the present EGM. The present EGM builds on the findings of the mega map which found that the evidence for protecting children from violence and exploitation is low in LMICs; nonetheless, it should be a priority area for policy and practice (UNICEF, 2018b).

There are additional related ongoing maps and published maps. There is a map on child maltreatment and neglect which is restricted to HICs (Axelsdottir, Biedilæ, & Albers, 2018). A second map for LMICs focuses on child neglect (Sinha, Radhika, Jha, & John, 2018) whereas the present map focuses on VAC (we elaborate on this distinction below). The third map (Albers, et al., 2019) is examining institutional responses to child maltreatment, not focusing on any specific region.

A fourth evidence map has been published in 2019 by the Institute for Security Studies (ISS) in partnership with the University of Johannesburg and Witwatersrand University to address violence against women and children in South Africa (Amisi, et al., 2019).

There is no existing EGM for interventions to address VAC in LMICs.

## 4 OBJECTIVES

### 4.1 Objectives

The objectives of this EGM are to:

i. Identify existing gaps in evidence to better inform future investment in research on interventions for VAC.
ii. Identify clusters of impact evaluation that offer opportunities for evidence synthesis on VAC.
iii. Identify, appraise and provide short summaries of existing evidence from systematic reviews and impact evaluations of the effects of interventions to reduce VAC.

## 5 METHODS

### 5.1 EGM: Definition and scope

#### 5.1.1 What is an EGM?

Mapping the evidence in an existing area is an approach that has been used since the early 2000s (Saran & White, 2018). EGMs are “evidence collections” (Snilstveit, Vojtkova, Bhavsar, & Gaarder, 2013) that provide a visual overview of the availability of evidence for a particular sector‐in this VAC.

The present EGM provides an overview of all available evidence on the key outcome categories and interventions aimed at reducing VAC in LMICs using an intervention‐outcome framework (in this case, the INSPIRE framework).

The EGM is presented in two dimensions: the rows list interventions and the column list outcome categories. Each cell shows studies that contain evidence on that combination of intervention and outcomes.

By providing an interactive and visual version of what we know, do not know, and not know enough, about a particular issue, EGMs may help inform research commissioning and fund allocations for further research.

#### 5.1.2 Scope

The scope of this EGM is to capture available effectiveness studies on reducing VAC in LMICs. We included studies assessing effectiveness of interventions to reduce victimisation as well studies assessing impacts of interventions on perpetration of VAC.

This map excludes self‐directed violence and child neglect, as this is the scope of an ongoing EGM on child abuse and neglect that includes self‐directed injuries in LMICs (Sinha et al., 2018). We also excluded structural and collective violence such as genocide, wars, political violence because the nature of effective interventions to combat collective violence are likely to be dependent on socio‐political context and factors and are thus considered to be outside the scope of the present EGM.

#### 5.1.3 Types of evidence

This EGM includes systematic reviews and impact evaluations on the effectiveness of interventions to prevent VAC.

Systematic reviews are eligible to be included if they contain one or more studies from LMICs. The key characteristics for a review to be included as a “systematic review”

1. A clearly stated set of objectives with pre‐defined eligibility criteria for studies.
2. An explicit, reproducible search strategy.
3. A systematic search that attempts to identify studies that would meet the eligibility criteria.
4. A systematic presentation, and synthesis, of the characteristics and findings of the included studies.

Among impact evaluation, we included RCTs; non‐experimental evaluations (controlled before–after studies, uncontrolled before–after and interrupted time‐series); regression discontinuity design (RDD); difference‐in‐difference (DID); and studies employing matching techniques such as propensity score matching (PSM). Relevant ongoing studies are also included. The detailed eligibility criteria are given in Supporting Information Appendix 3. We did not include qualitative research studies as per the scope of this EGM.

#### 5.1.4 Type of population

As defined in the INSPIRE handbook, “Child” means any person aged under 18 years (WHO, 2018). Following this guideline, interventions regarding children irrespective of their sex in the age group of less than or equal to 18 years in LMICs are included in the EGM.

The age group is classified based on the WHO age criteria stated as follows: Infanthood (<3 years of age), childhood (3–10 years), adolescence (10–18 years) (WHO, 2017).

The population subgroup of interest includes:

- Target group of interventions: Women and girls, children with disabilities, children belonging to ethnic minorities, child sex workers, child brides, isolated children/street children, children with HIV/AIDS, migrants and children affected by conflict and humanitarian crises, parents/caregivers and teachers.
- Perpetration of violence: Peers, Parents, Romantic partner/intimate partner, teachers and other

#### 5.1.5 Types of interventions

Table 2 lists the intervention categories and subcategories. Examples of programme names are given in brackets. These are listed to aid with search and coding and did not appear in the subcategory label in the map.

**Table 2:** Intervention categories, subcategories and examples

Intervention category	Subcategory	Examples
1. Laws, crime and justice	Law	Laws banning corporal punishment or increasing legal consequences for its perpetration, laws criminalizing or increasing legal consequences for perpetration of sexual abuse and exploitation of children, laws preventing or reducing substance misuse (advertisement, prices, coupons), laws limiting youth access to firearms and other weapons and engagement in conflict, family law/child protection legislations, law on Violence against children, law on media content regulation
	Crime and justice systems	Treatment programmes and other safeguards for juvenile offenders in the crime and justice system/gangs, strengthening police and judicial systems for child protection, increasing access to informal justice, including community‐based legal aid and paralegal programmes, adolescent intimate partner violence and dating violence
2. Norms and values	Community mobilisation programmes	Community‐wide interventions to raise awareness of child violence
	Bystander interventions	Interventions to empower bystanders to intervene and prevent violence
	Media campaigns including mass media and education	Media campaigns, edutainment highlighting the issue of child violence
3. Safe environments	Making existing environments safe	Reducing violence by addressing “hotspots”, interrupting the spread of violence, Improving the built environment (safe homes, schools), urban upgrading, Zoning strategies to reduce violence, child protective services including safe orphanages/homes for children without guardianship
	Creating safe places	School WASH and infrastructure
4. Parent, child and caregiver support	Parent‐training and education—interventions that promote positive parenting practices	Parent and child support groups, government agencies that coordinate/streamline all activities related to parenting and parent support, home visiting programmes, group parenting programmes, integrated parenting programmes
	Maternal and paternal mental health	Counselling and therapy for mental health support
	Peer‐relationship training	Peer training, peer educators
5. Income and economic strengthening	Economic transfers	Conditional cash transfers, unconditional cash transfers, public works or cash‐for‐work, In‐kind transfers (food, vouchers, assets).
	Income generating or savings/credit interventions	Group saving and loans (with and without additional components, e.g., gender equity training), microfinance or credit (with and without additional components, e.g., gender norms training), financial inclusion programmes (including savings programmes, financial literacy, access to banking), livelihood or agricultural productivity programmes (including graduation programmes), skills training/vocational or entrepreneurship programmes
	Broad‐based social protection	Health and other insurance, employer and labour force benefits (including unemployment befits, maternity leave policies), pensions and retirement benefits, disability benefits
6. Response and support services	Counselling and therapeutic approaches	Specialised counselling and therapeutic services for victims of violence
	Screening and training	Reporting combined with interventions: training the health professional/social workers/teachers to identify possible exposure or risk of exposure to violence
	Children in care	Includes alternative family care (foster or kinship care) or institutional care (orphanages, group homes, juvenile detention centres, or residential treatment centres) interventions involving social welfare services, Shelters and crisis centres
	Media and communication	Awareness on access to services/reporting
7. Education and life skills	Gender transformative approaches	Including sexual and reproductive health education
	Life and social skills training	Violence prevention, bullying prevention programmes, self‐defence, adolescent intimate partner violence/dating violence prevention (interventions to prevent abusive behaviour in adolescent peer relationships)

The categories of interventions are largely based on INSPIRE guidelines. The subcategories are based on pilot coding of 30 key studies, expert advice and discussion within the team and advisory group members. Definitions for intervention subcategories are given in Supporting Information Appendix 1.

The intervention categories included in our map are based on the INSPIRE guidelines are:

1. Laws, crime and justice
2. Norms and values
3. Safe environments
4. Parent, child and caregiver support
5. Income and economic strengthening
6. Response and support services
7. Education and skills

#### 5.1.6 Types of outcome categories

The seven main outcome categories are listed in Table 3.

**Table 3:** Outcome categories and subcategories

Outcome categories	Subcategories
1. Violence	Sexual
	Physical
	Emotional/psychological
2. Norms and values	Parenting beliefs and practices
	Gender roles, attitudes and social norms
	Delinquent, violent and other risk‐taking behaviour (including reoffending, recidivism)
	Empowerment
3. Health	Substance abuse
	Child development and child mental health
	Maternal and paternal mental health
	Morbidity and mortality
	Sexual and reproductive health
4. Safety and risk factors for other harms	Social isolation (homeless and street‐connected children)
	Female genital mutilation (FGM) and child marriage
	Child labour and child trafficking
	Safe environment/spaces
5. Economic and social	Poverty and food security
	Employment and labour force participation
	Savings and credit
	Social inclusion and gender equity
	Social discrimination
6. Education	School enrolment and attendance
	School performance
	WASH and infrastructure
	Gender roles and life skills
	Gender roles and life skills
7. Cost‐analysis	Cost‐effectiveness
	Cost‐benefit

#### 5.1.7 Types of location/situation

The primary population of interest for this map is children and adolescents from LMICs. LMICs are defined by World Bank as:

- low‐income economies—those with a Gross National Income (GNI) less than $995;
- lower‐middle‐income economies—those with a GNI per capita between $996 and $3,895; and
- upper‐middle‐income economies—those with a GNI per capita between $3,896 and $12,055 (World Bank, 2018b).

Studies with multiple populations are included in the map, if they include children of or under 18 years. Systematic reviews with a global focus are included if they have atleast one impact evaluations conducted in LMICs included in the systematic review.

#### 5.1.8 Types of settings

The EGM includes all types of settings where interventions for VAC were implemented. Based on the framework, the settings include school, home, centre or facility, community, etc. We did not exclude any study based on setting.

#### 5.1.9 Search methods and sources

The search for the EGM was conducted online in two stages:

The EGM search method has been developed to scan literature in two phases:

**Phase 1 (2019–2020)**: We validated the screening and coding tool through pilot screening and coding from the studies included in the INSPIRE Seven Strategies for Ending Violence against Children (WHO, 2016b).

The next step included a search of relevant systematic reviews and impact evaluation studies from academic and grey literature searches from the year January 2000–December 2019.

a. Academic search: An experienced systematic reviewer drafted the search strategy, validated by an experienced information specialist. The search strategy consisted of range of search concepts and associated terms. The search strategy was applied to systematic review databases, such as 3ie and Campbell Collaboration databases; academic databases, such as OVID and Embase (Table 1).
  In total, 2862 records were identified from the search of academic database.
b. Limited grey literature search: This included studies from non‐academic sources such as:
  ➣ Organisational websites: such as UNICEF, DFID, WHO, USAID, Action Aid etc. (Table 1).

A total of 185 additional studies were identified from the grey literature search. The final number of included studies, after title and abstract and full text screening, were 152. Reference checking was performed for these 152 studies.

Expert consultation: An expert advisory group was established and consulted at every stage of the EGM development, from coding development to analysis of findings including search resources and studies.

**Phase 2** is underway and extends the searches to Arabic, Chinese, French, Portuguese and Spanish.

#### 5.1.10 Protocol

The EGM protocol was published on Wiley Online Library (5th September 2019) and is available at https://onlinelibrary.wiley.com/doi/full/10.1002/cl2.1040.

### 5.2 Stakeholder engagement

UNICEF Office of Research–Innocenti (UNICEF Innocenti), commissioned this map. They will take the lead in introducing the map into relevant policy discussions including launching it with the Global Partnership to End Violence against Children as part of a series of global public resources that can be widely shared and used.

An advisory group was formed at the inception stage of this EGM. Feedback from the group was received at all stages of the EGM to review and comment on interventions, studies, outputs, map findings and provide advice on dissemination channels.

The advisory group members for this EGM includes experts representing all of the categories of the INSPIRE framework on social, economic, education, health and wellbeing, who have been involved in working towards reducing/ending VAC in their own fields. Through a joint effort by UNICEF Innocenti and the advisory group members, this work will contribute to greater alignment of global and national efforts and will form the basis for improving the evidence base on VAC in LMICs.

Advisory group members:

1. Dr. Karen Devries, Associate Professor in Social Epidemiology, London School of Hygiene and Tropical Medicine
2. Professor John Lawrence Aber, Willner Family Professor in Psychology and Public Policy University Professor, New York University
3. Professor Lorraine Sherr, Head of Health Psychology Unit, Institute of Global Health, University College London
4. Shivit Bakrania, Knowledge Management Specialist, Research Facilitation and Knowledge Management unit, UNICEF Office of Research–Innocenti
5. Professor A.K. Shiva Kumar, Global Co‐Chair, Know Violence in Childhood
6. Dr. Andrés Villaveces, Senior Scientist, Division of Violence Prevention, NCIPC, US Centers for Disease Control and Prevention, and Department of Epidemiology, University of North Carolina (UNC), Chapel Hill
7. Dr. Amber Peterman, Consultant, UNICEF Innocenti and Associate Professor at UNC Chapel Hill
8. Professor Lucie Cluver, Professor of Child and Family Social Work, Centre for Evidence‐Based Social Intervention in the Department of Social Policy and Intervention, Oxford University
9. Professor Rebecka Lundgren, Infectious Diseases & Global Public Health Center on Gender Equity and Health, University of California San Diego
10. Dr. Charlene Coore Desai, Resident Advisor, USAID Applying Science to Strengthen and Improve Systems (ASSIST) Project, Jamaica
11. Kerry Albright, Chief, Research Facilitation and Knowledge Management unit, UNICEF Office of Research–Innocenti

Additionally, comments were received on the final report from Alessandra Guedes and Stephen Blight (UNICEF) and Catherine Maternowska, (Global Partnership to End Violence Against Children).

## 6 DIMENSIONS OF THE MAP

### 6.1 Description of Intervention

Table 2 lists the intervention categories that are largely based on INSPIRE categories. The subcategories have been modified to incorporate broad interventions groups.

### 6.2 Description of outcome categories

Included studies were those that reported interventions for different types and forms of VAC. Studies were categorised by seven outcome categories on which they reported: direct impact on violence, norms and values, economic and social factors, safety and risk factors for other harms, health and education outcomes (Box 4). Additionally, the report assesses the availability of information on cost‐analysis as a seventh outcome category.

Box 4 **Distribution of studies from Africa**

**Table:** 

South Africa: Five (5) studies from South Africa relate to parenting interventions to reduce child abuse. The other studies from South Africa include microfinance interventions (2) and gender transformative approaches to reduce violence (2) and an assessment of the effectiveness of cash transfers on child labor (1).
Uganda: The majority of the studies from Uganda are on skills training and microfinance to reduce VAC. The other studies from Uganda include interventions such as gender‐transformative approaches (1), parenting interventions (1) and interventions for teachers to reduce violence in schools (1).
Kenya: The impact evaluations from Kenya, the country with the third highest number of intervention studies in Sub‐Saharan Africa, include interventions with gender transformative approaches to reduction of all forms of violence against children (2), economic transfers and parenting interventions (1), reduction of female genital cutting (1) and child marriage (1).

The outcome categories and subcategories were mapped across the intervention categories and subcategories in the present map. The seven main outcome categories are listed in Table 3.

### 6.3 Description of population

Children irrespective of their sex in the age group of less than or equal to 18 years were included in the EGM.

1. Target group of interventions: Women and girls, children with disabilities, children belonging to ethnic minorities, child sex workers, child brides, isolated children/street children, children with HIV/AIDS, migrants and children affected by conflict and humanitarian crises, parents/caregivers and teachers.
2. Perpetration of violence: Peers, Parents, Romantic partner/intimate partner, teachers and other.

### 6.4 Description of geographic location

Studies from low‐income countries, LMICs and upper‐middle‐income countries are included in the present map.

The category LMIC is defined by (World Bank, 2018b) as:

- low‐income economies—those with a Gross National Income (GNI) less than $995;
- lower‐middle‐income economies—those with a GNI per capita between $996 and $3,895; and
- upper‐middle‐income economies—those with a GNI per capita between $3,896 and $12,055.

There are 47 countries in the LMICs criteria defined by The World Bank globally.

Global systematic reviews were included if they contained at least one impact evaluation conducted in LMICs. Impact evaluation and systematic reviews from HICs alone were excluded.

### 6.5 Analysis and presentation

#### 6.5.1 Presentation

The EGM has traditionally two primary dimensions intervention as rows and outcomes as columns. In the online interactive map, we used the additional dimensions as filters:

1. Target group of interventions: Women and girls, children with disabilities, children belonging to ethnic minorities, child sex workers, child brides, isolated children/street children, children with HIV/AIDS, migrants and children affected by conflict and humanitarian crises, parents/caregivers and teachers.
2. Perpetration of violence: Peers, Parents, Romantic partner/intimate partner, teachers and other
3. Region: East Asia & Pacific, Europe & Central Asia, Latin America & Caribbean, Middle East & North Africa, North America, South Asia, Sub‐Saharan Africa
4. Country
5. Economies: Low‐income economies, Lower‐middle‐income economies, Upper‐middle‐income economies, High‐income economies
6. Forms of violence:
  - Intimate partner violence
  - Peer violence
  - Corporal punishment
  - Unclassified category (Here we include studies evaluating interventions that do not specify a type or form of violence, or which report more than one form of violence, or which cover broader harms experienced by children, or which include attention to the consequences of exposure to violence, such as, for example, an intervention studying impact of counselling to empower sexually abused girls through cognitive behavioural group therapy training which is categorised here)
7. Fragile and conflict‐affected regions (FCS): defined based list of conflict‐affected regions updated as per year 2018–2019 (World Bank, 2018b).

For this map we will present *three forms of visualisation* of evidence (Snapshots for each added as annex)

1. Interventions as rows and outcomes as columns.
2. Interventions as rows and additional filters; forms of violence and perpetrator of violence as columns.
3. Interventions as rows and additional filter; regions as columns

#### 6.5.2 Dependency

Each entry in the map is a systematic review or impact evaluation of effectiveness. Each item in the map reports one study, as we did not come across any review of reviews. The readers must be aware that though the representation of studies is separate (for systematic reviews and impact evaluations), there is an overlap of evidence as one study can be represented in more than one cell, if the study dealt with multiple interventions or outcomes.

## 7 DATA COLLECTION AND ANALYSIS

### 7.1 Screening and study selection

The screening of the studies for inclusion or exclusion in the map was conducted in two stages. The first stage involved title and abstract screening. The second stage involved screening the full text of the articles included at the first stage. Screening was done by two independent reviewers (P. P. and J. A.) using predefined eligibility criteria (given in Supporting Information Appendix 3). A third reviewer (A. S.) resolved any disagreements in screening after a discussion with both of the initial reviewers. EPPI Reviewer 4 is the online software (Thomas, Brunton, & Graziosi, 2010) used for screening and coding. The screening tool has been added as Supporting Information Appendix 4. We did not use automation at any point during the screening or coding process.

#### 7.1.1 Stage 1: Title and abstract screening

We uploaded search results from databases to EPPI Reviewer 4 software for screening. In order to pass stage one, the title or abstract had to meet the eligibility criteria as listed in Supporting Information Supporting Information Appendix 3.

#### 7.1.2 Stage 2: Full text screening

Full text documents were retrieved for all documents that passed stage one. Two reviewers independently evaluated all studies. Studies had to meet all of the inclusion/exclusion criteria set out previously in order to advance to full review.

### 7.2 Data extraction and management

We used a standardised data extraction form (presented in Supporting Information Appendix 5) to extract descriptive data from all studies meeting the eligibility criteria.

Each included study was coded independently by a team of two coders (J. A. and P.P.) using the coding tool (Supporting Information Appendix 5) covering study characteristics, population, interventions, outcomes, region, countries and type of violence. A third reviewer (A. S.) resolved the conflicts. We contacted the authors for the articles that we could not retrieve. If we did not receive the reply within 15 days, we kept such studies under “awaiting classification”. A reference list of the included studies and systematic reviews were compiled by A. S., P. P., and J. A.

Two authors (H. W. and R. S.) validated coding and reporting through random coding checks for a portion of studies.

### 7.3 Tools for assessing risk of bias/critical appraisal of included reviews

#### 7.3.1 Critical appraisal

We critically appraised both systematic reviews and impact evaluations to indicate confidence in study findings.

##### Systematic reviews

For systematic reviews we scored each study using the 16‐item checklist, AMSTAR 2—“Assessing the Methodological Confidence of Systematic Reviews” (Shea, et al., 2017) (Supporting Information Appendix 6).

The 16 items cover:

(1) population, interventions and outcomes in inclusion criteria,
(2) ex ante protocol,
(3) rationale for included study designs,
(4) comprehensive literature search,
(5) duplicate screening,
(6) duplicate data extraction,
(7) list of excluded studies with justification,
(8) adequate description of included studies,
(9) adequate risk of bias assessment,
(10) report sources of funding,
(11) appropriate use of meta‐analysis,
(12) risk of bias assessment for meta‐analysis,
(13) allowance for risk of bias in discussing findings,
(14) analysis of heterogeneity,
(15) analysis of publication bias, and
(16) report conflicts of interest.

Seven of these criteria have been applied as relevant in our critical assessment. Items 2, 4, 7, 9, 11, 13 and 15 are considered “critical”. Study confidence is rated “high” if there is no more than one non‐critical weakness, and medium if there is no critical weakness but more than one non‐ critical weakness. Studies with one or more critical weaknesses are rated “low” confidence.

##### Impact Evaluations

We used a modified risk of bias tool (Supporting Information Appendix 7) to rate the confidence of impact evaluations.

**Figure 1:**
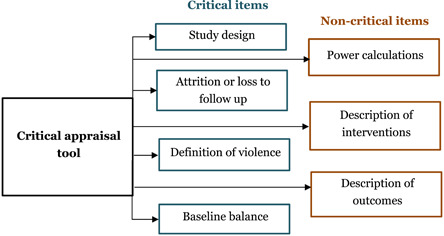
Graphical representation of critical appraisal tool for impact evaluations

This tool includes six criteria that are appropriate for the assessment of quantitative impact evaluations. These are as follows:

1. *Study design (potential confounders taken into account)*: Impact evaluations need either a well‐designed control group (preferably based on random assignment) or an estimation technique that controls for confounding and the associated possibility of selection bias.
2. *Power calculations*: Small sample sizes can result in an underpowered study with a high risk of not detecting an effect from the intervention when there actually is one. The combination of underpowered studies and publication bias can put an upward bias on the assessment of the overall effect size from a body of literature. Power calculation helps to address the problem of sample size before the study to determine the required sample size. We have not used this item in the overall assessment of the study. However, coding a mention of power calculations signals the importance of both conducting and reporting power calculations.
3. *Attrition or losses to follow‐up*: can be a major source of bias in studies, especially if there is differential attrition between the treatment and comparison group (called the control group in the case of RCTs) so that the two may no longer be balanced in pre‐intervention characteristics. The US Institute of Education Sciences What Works Clearing House (WWC) has developed standards for acceptable levels of attrition, in aggregate and the differential.
4. *Clear definition of violence*: For a study to be useful, the study population must be clear, which means that the type and degree of violence must be clearly defined, preferably with reference to a widely used international standard.
5. *Description of intervention*: If the intervention is not well described then the evidence may be misinterpreted to support an intervention that is not actually supported by study findings. For example, “case work” or “shelter” are very broad descriptions, so more detail of the intervention is required so as to know what is actually being evaluated. We rate as low if the description is just named with no description, medium if there is a short description, and high if there is a detailed description. We do not use this item in the overall assessment of the study.
6. *Definition of outcomes*: Outcomes should be clearly defined so that study findings can be properly interpreted. As far as possible, unless a subjective perception is required, questions should rely on objective factors, and utilise data collection instruments which have been validated for the context in which they are being applied. We rate as high if there is clear definition of the outcome and how it is being measured, or reference to an existing tool. Medium rating is given if there is a brief description, and low if the outcome is named but not adequately described.
7. *Baseline balance*: Baseline balance means that the treatment and comparison groups have the same average characteristics at baseline, not only for outcomes but other factors which may affect the impact of the programme such as a prior history of parental alcohol abuse. We rate low confidence in study findings if baseline balance is not reported for non‐RCTs or it is reported and there is a significant difference of 10 or more than 10%, medium confidence if imbalance is between 5% and 10%, and high if an RCT or if imbalance is 5 or less than 5% (Figure 1).

8. *Overall assessment*: The overall assessment uses a “weakest link in the chain” principle so that the overall assessment is the lowest of assessment given to any of the relevant items. As mentioned above, not all items are used in this assessment. So the overall assessment, is the lowest of the assessments for Items 1, 4, 6 and 7.

##### Ethical considerations

Many difficult ethical dilemmas may arise when gathering information about children and adolescents. Given the obviously sensitive nature of research into VAC, it is indeed challenging to conduct such studies. The foundational commitment to “do no harm” that underpins all child protection work necessitates a central focus on ensuring the safety of respondents and appropriate and effective referral services, in line with the duty of care towards all children (UNICEF, 2018a). Further, every person has a right to privacy; hence preserving the confidentiality of personal information is one of the fundamental principles governing the collection of data about individuals. In line with the above stated challenges, an ethical adequacy tool for measuring the ethical standards of primary intervention/effects study was developed (Council for International Organizations of Medical Sciences, 2009; Population Council, 2005; Save the Children, 2004; WHO, 2007); UNICEF's child‐specific guidance for ethical evidence generation) for the impact evaluations included in this EGM.

The nine‐item tool had five critical and four non‐critical items. The scoring identified “Strong ethical standards”, “Moderate ethical standards” and “Weak ethical standards”. The complete tool with scoring can be found in the Supporting Information Appendix 8. We did not disqualify any study based on the adequacy of ethical standards.

We note that a uniformly used and standardised ethical appraisal tool does not exist for VAC studies that explicitly mention points on privacy, referral, adverse event protocol and informed consent.

## 8 RESULTS

### 8.1 Description of studies

#### 8.1.1 Results of the search

**Figure 2:**
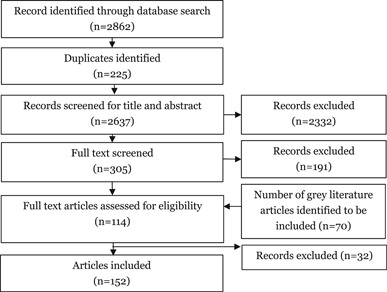
PRISMA for VAC evidence and gap map. VAC, violence against children

The screening process (Figure 2) resulted in 152 records to be included in Phase 1. Of these, 55 were systematic reviews and 97 were impact evaluations (see list of included studies in “References”). The search is to be extended to other languages, including, Arabic, Chinese, French, Portuguese and Spanish, in Phase 2.

Figure 2 provides an overview of the results of the search and screening process to identify studies for inclusion. The search returned 2,862 records from database searches with 2,637 studies remaining for screening at title and abstract after duplicate removal. We extended the search to some of the key websites and online grey literature sources as mentioned in the previous section on methodology.

As a result of T&A screening, 305 studies were reviewed for full text. Of these 152 studies met the inclusion criteria for the EGM. The main reasons for exclusion were lack of relevant interventions and study methodology. Due to the large number of studies screened at full text, we do not provide a full list of excluded studies here, but this list is available upon request.

### 8.2 Description of included studies

There are many more entries in the map than there are studies. This is because the studies, especially systematic reviews, were coded and mapped for all the intervention categories and subcategories that they included. The results present the findings from the 152 included studies (55 systematic reviews and 97 impact evaluations).

### 8.3 Aggregate map

Table 4 shows the aggregate maps of interventions and outcomes.

**Table 4:** Aggregate map, number of studies by intervention category and outcome category

	Violence	Norms and values	Health	Safety and risk factor for harms	Economic and social	Cost‐analysis	Education
Laws, crime and justice	5	4	2	2	1	0	0
Norms and values	42	25	13	20	4	0	11
Safe environment	13	7	4	10	2	0	4
Parent, child and caregiver support	48	26	20	11	8	0	5
Income and economic strengthening	47	21	17	37	18	2	31
Response and support services	36	8	16	12	0	0	2
Education and life skills	58	34	23	24	7	1	20

Populated cells: These subcategories for interventions and subcategories provide the most heavily populated cells on the map:

- The impact of education and life skills on direct impact on violence (58) and norms and values (34). There is a particularly large evidence base for income and economic strengthening on violence (47), safety and risk factor for harms (37) and education (31).
- The impact of parent, child and caregiver support interventions on violence (48) and norms and values (26).

#### 8.3.1 Laws, crime and justice

- This is the least populated sector with only five studies identified.
- Most common outcomes reported were direct impact on VAC (5) and impact on changing norms and values (4).

#### 8.3.2 Norms and values

- Thirty‐five (35) out of 43 studies for norms and values interventions focused on community mobilisation programmes.
- Fourteen (14) studies were identified on media campaign.
- Limited evidence was found on the effectiveness of bystander interventions (4).
- On the outcomes front, 26 studies reported impact on changing norms and values such as change in gender roles (21), empowerment (5) and belief in parenting practices (3).
- There are other outcomes reported as well, such as safety and risk factors for other harms (25), direct reduction of violence (47) and impact on health (19).

#### 8.3.3 Safe environment

- This sector has limited evidence with only 15 studies, of which nine studies were identified on interventions for creating safe places and six on making existing environment safe.
- On the outcomes front, there are 13 studies reporting direct impact on violence, 11 studies reporting impact on safety and risk factors for other harms and 7 on changing norms and values.

#### 8.3.4 Parent, child and caregiver support

- Parent, child and caregiver support includes 48 studies, 39 of which focused on parent training and education and 10 on peer‐relationship and training.
- On the outcomes front, we identified only 4 studies that assessed the impact on maternal and paternal mental health.
- Reduction in VAC (53) is the most commonly reported outcome, followed by change in norms and values (29), impact on health outcomes (23) such as, sexual and reproductive health (12), child mental health (11), mental/paternal mental health (7) and substance abuse (4).

#### 8.3.5 Income and economic strengthening

- The intervention area income and economic strengthening has a total of 53 studies. Majority of studies were found assessing the effectiveness of economic transfers (40), followed by income generation, savings or credit interventions (16).
- Insurance and welfare schemes is least populated with only five (5) studies.
- On the outcomes front, safety and risk factors for other harms (46) is the most commonly reported outcome after reduction in violence (49). Safety and risk factor for other harms includes outcome like reduction in child labour and trafficking (29); female genital mutilation and child marriage (18).
- Second common outcome reported is the impact on economic and social outcomes (19) such as employment and labour force participation (7), savings and credit (9) and poverty and food security (5).

#### 8.3.6 Response and support services

- Thirty‐six studies were identified related to the intervention area response and support services, of which 21 related to screening and training and 17 to counselling and therapeutic approaches.
- There are stark gaps in the evidence identified on the effectiveness of interventions for children in care (3) and dissemination of information by media and communication (2).
- The most common outcomes reported as a result of response and support interventions are impact on violence (44) and health outcomes (23).

#### 8.3.7 Education and life skills

- The intervention area education and life skills—which maps the studies of effect of educational and school‐based interventions on VAC is the most heavily concentrated (60) (Table 4).
- Under education interventions, gender transformative approaches and life and social skills are almost equally concentrated with 41 and 32 studies, respectively.
- On the outcomes front, 24 studies reported impact on educational outcomes as a result of the interventions.
- Most commonly reported outcomes as result of interventions in this area were found influencing norms and values (37), followed by impact on health (26) and safety and risk factors for other harms (26).

### 8.4 Synthesis of included studies by primary dimension (intervention‐outcome)

#### 8.4.1 Number of studies by intervention categories

##### Systematic reviews

**Figure 3:**
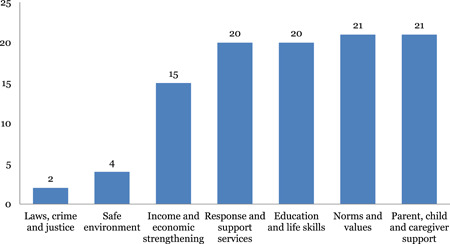
Number of systematic reviews by intervention categories

Figure 3 shows the number of systematic reviews by intervention categories. Laws, crime and justice (2) and safe environment (4) are the least populated areas of the map. We identified an almost equal number of systematic reviews across the categories of parent, child and caregiver support (21); norms and values (21), response and support services (20) and education and life skills (20).

##### Impact evaluation

**Figure 4:**
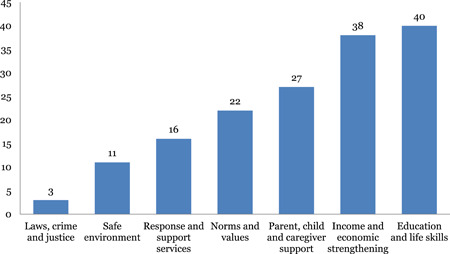
Number of impact evaluations by intervention

As with systematic reviews, laws, crime and justice (3) and safe environment (11) are the least populated areas for impact evaluation as well. Education and life skills (40) and income and economic strengthening (38) are the most concentrated (Figure 4).

##### Impact evaluations by intervention and study design

**Figure 5:**
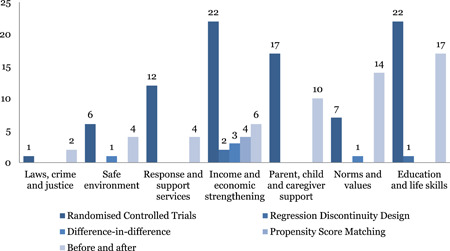
Number of impact evaluations by intervention and study design

RCTs account for close to half of the impact evaluations (51 RCTs out of 91) being particularly prominent in the intervention areas education and life skills (19), norms and values (16) and parent, child and caregiver support (10) (Figure 5).

##### Impact evaluations by intervention area and type of violence

**Figure 6:**
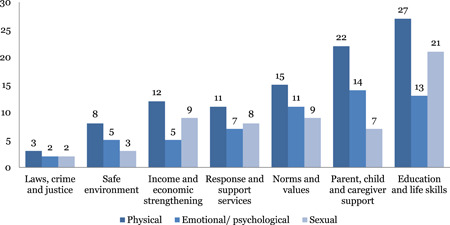
Impact evaluations by intervention and type of violence

Physical violence is the most common type of violence studied across the various intervention categories (Figure 6).

##### Fine‐grained analysis by intervention subcategory

Further fine‐grained analysis reveals that though there is a fair amount of evidence on parent, child and caregiver support, evidence is limited on children in care (3) and media and communication interventions (2). There are only five (5) studies on substance abuse and five (5) studies on morbidity and mortality as outcomes.

There are also limited studies on insurance and welfare schemes assessing their impact on the reduction of violence in children (5).

Empty reviews are systematic reviews with no included eligible studies in a particular sector. There were two empty reviews included in the present EGM: the first, Parker & Turner (2014) was a Cochrane review on psychoanalytic psychotherapy for sexually abused children and adolescents, and received no financial support. The second systematic review by Adelufosi, Arikpo, Aquaisua & Meremikwu (2017) was on cognitive behaviour therapy for treating depression in cases with female genital mutilation, and was financially supported by WHO Department of Reproductive Health and Research.

#### 8.4.2 Number of studies by outcome category

##### Systematic reviews

**Figure 7:**
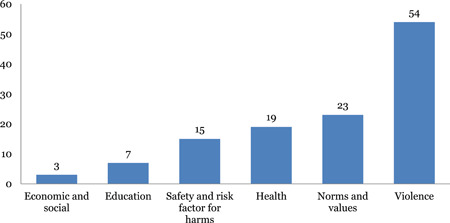
Number of systematic reviews by outcome categories

Figure 7 shows systematic reviews by outcome area. Direct impact on VAC is the most studied outcome (54) followed by influence on norms and values (22) and impact on health (19).

##### Impact evaluations

**Figure 8:**
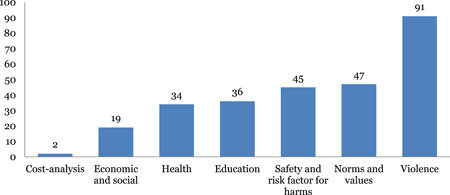
Number of impact evaluations by outcome category

As with systematic reviews, direct impact on VAC was the most reported outcome (91); however, we also found studies reporting impact on changing norms and values (47), safety and risk factors for other harms (45), education (36) and health (34). We found few impact evaluations conducted in relation to cost‐analysis (2), and relatively few studies assessed economic and social outcomes (19) (Figure 8).

##### Number of impact evaluations by types of violence

**Figure 9:**
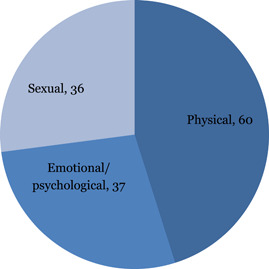
Impact evaluations by type of violence

Physical violence is the most studied type of violence (60) with equal number of studies on sexual (37) and emotional violence (36) (Figure 9).

### 8.5 Description of included studies by secondary dimensions (filters)

#### 8.5.1 Evidence by region and country

##### Impact evaluations

**Figure 10:**
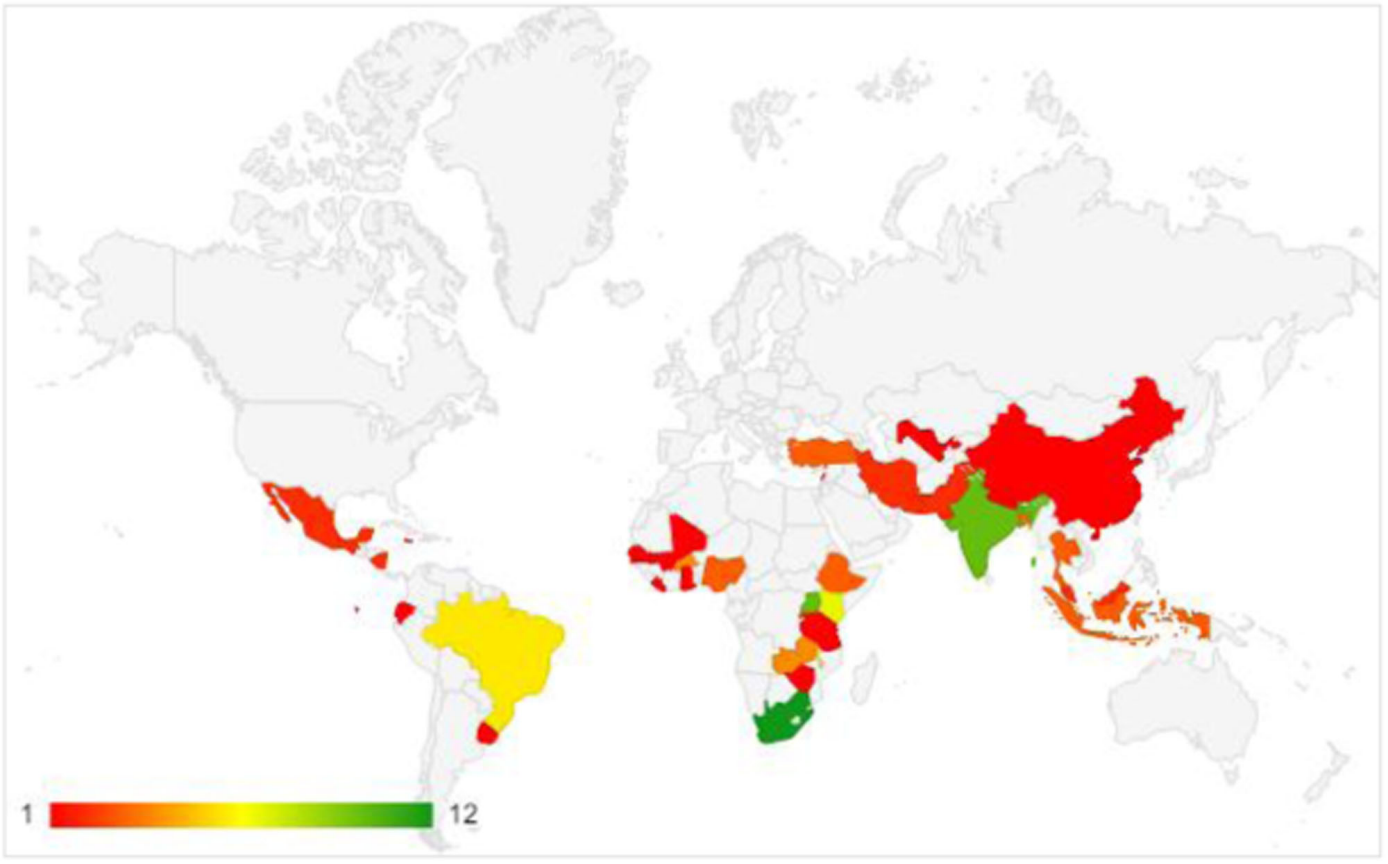
Geographic heat map of impact evaluations included in the EGM. EGM, evidence and gap map

Figure 10 shows that the majority of impact evaluations come from Sub‐Saharan Africa (59).

South Africa (13) is the country with the highest number of impact evaluations. Uganda (10) was the country with the second‐highest number of impact evaluations in Sub‐Saharan Africa, followed by Kenya (6). The greatest number of studies from the Sub‐Saharan African region were on economic transfers (10), followed by parenting programmes (7) and gender transformative approaches (6) to reduce child abuse.

Thirteen impact evaluations come from South Asia, of which 8 are from India, 3 from Bangladesh and 1 each from Pakistan and Nepal.

Thirteen impact evaluations come from South Asia, of which 8 are from India, 3 from Bangladesh and 1 each from Pakistan and Nepal.

Eleven from Latin America and the Caribbean, eight (8) from East Asia and Pacific, only four (4) studies from the Middle East and North Africa and three (3) from Europe and Central Asia.

##### Systematic reviews

**Figure 11:**
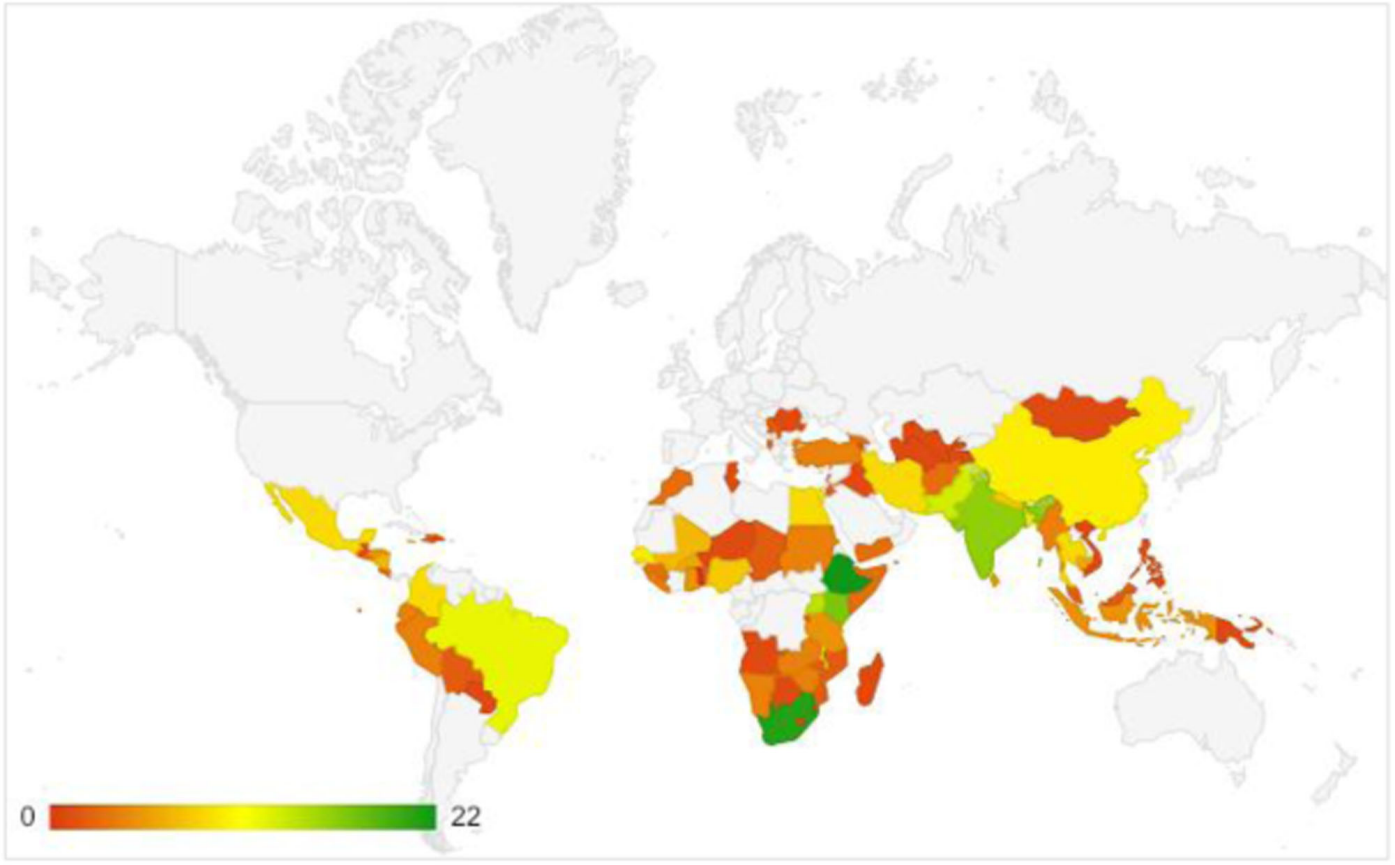
Geographic heat map of systematic reviews included in the EGM. EGM, evidence and gap map

**Figure 12:**
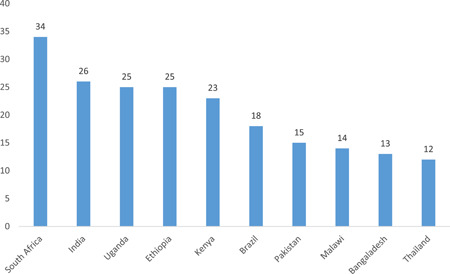
Countries with the largest number of combined studies (impact evaluations and systematic reviews)

This pattern remains similar for systematic reviews with a concentration in Sub‐Saharan Africa (36), South Asia (27), East Asia and Pacific (28) and Latin America and Caribbean (24) as shown in Figure 11 and Figure 12.

##### By income group

Table 3 gives the list of countries and the frequency of studies from these countries. Twelve impact evaluation studies in the EGM are from South Africa and 10 are from India. Uganda and Kenya also recorded a high number of impact evaluations (Table 5).

**Table 5:** Number of impact evaluation (*n*) by countries (World Bank, 2018b)

Low‐income countries	Low and middle income	Upper middle income
Uganda (10)	India (10)	South Africa (12)
Malawi (5)	Kenya (7)	Brazil (6)
Burkina Faso (4)	Zambia (4)	Thailand (3)
Ethiopia (3)	Indonesia (3)	Turkey (3)
Rwanda (2)	Nigeria (3)	Mexico (2)
Congo (1)	Bangladesh (3)	Malaysia (2)
Liberia (1)	Côte d'Ivoire (2)	Iran (2)
Mali (1)	Pakistan (2)	Lebanon (1)
Tanzania (1)	Nicaragua (2)	Ecuador (1)
Nepal (1)	Zimbabwe (1)	Jamaica (1)
Nepal (1)	Senegal (1)	China (1)
	Ghana (1)	

##### By study design

In terms of methodology, a fair number of impact evaluations (45) had evidence from RCTs, a common impact evaluation method. As shown in Table 5, other common methodologies were quasi‐experimental—before and after design (35), matching/PSM methods (4 studies), 3 used DIDs and 3 used RDDs given in Table 6.

**Table 6:** Evidence by methodology

Impact evaluation design	Number of studies
RCT	51
Regression discontinuity design	3
Difference in difference	4
Propensity score matching	4
Quasi‐experimental (uncontrolled before after and controlled before after)	35

Out of the 35 quasi‐experimental studies, 10 studies used uncontrolled before–after design while the 25 studies used a controlled before–after design. Controlled before–after study designs are more reliable in terms of measuring effectiveness of interventions. As uncontrolled before–after designs are used in the absence of a control group, they are considered to provide lower confidence evidence on the effectiveness of interventions.

##### By forms of violence

Twenty‐one impact (21) evaluations assessed the impact of interventions specifically on intimate partner violence, followed by corporal punishment (10), and peer violence (7). Remaining studies could not be specifically classified by form of violence addressed, and are hence presented as a general, “unclassified” category.

##### For perpetration of violence

We found only a few studies assessing the impact of interventions on violence perpetration; 13 studies reported on parents/caregivers as perpetrators, 11 on romantic partner and intimate partners, 9 on peers and only 4 studies assessed interventions for teachers, clearly classified as perpetrators.

##### By Target group of interventions

**Figure 13:**
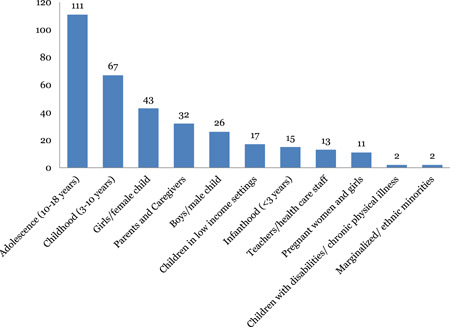
Population groups in the studies

Figure 13 gives the population group frequencies. Studies focusing on interventions to reduce violence amongst among adolescents or its impact on adolescent populations are the highest number (111), followed by children in the age group of 3–10 years (67). Studies assessing the impact of these interventions on children less than three years are sparse (15). A possible explanation is that studies on young children (less than 3 years) may be classified as neglect and child neglect is beyond the scope of this EGM.

##### Conflict‐affected and fragile countries

Analysis was undertaken according to a list of conflict‐affected and fragile regions and countries (WHO, 2018a) (the list available when this EGM was conducted). Studies from high‐, medium‐ and low‐fragility countries and their neighbours are given in Table 7.

**Table 7:** Number of included studies (impact evaluations and systematic reviews) from conflict‐affected and fragile countries

High fragility (48)	Medium fragility (38)	Low fragility (86)	Neighbours (44)
Pakistan (13)	Iran (11)	Ethiopia (26)	Uganda (24)
Nigeria (11)	Egypt (9)	Kenya (21)	Zambia (8)
DRC (6)	Zimbabwe (6)	Bangladesh (12)	Rwanda (4)
Afghanistan (3)	Guinea (3)	Mali (8)	Tanzania (3)
Somalia (3)	Haiti (2)	Nepal (8)	Tanzania (3)
South Sudan (3)	Tajikistan (1)	Liberia (5)	Benin (2)
Yemen (3)	Lebanon (1)	Honduras (3)	
Chad (2)	Congo (1)	Angola (1)	
Eritrea (1)	Uzbekistan (1)	Kyrgyz Republic (1)	
Libya (1)	Congo (1)	Madagascar (1)	
Myanmar (1)	Turkmenistan (1)		
Syria (1)	Turkmenistan (1)		

The greatest number of studies from high fragility contexts were from Pakistan (13) followed by Nigeria (11). For medium‐fragility contexts, the greatest number of studies were from Iran (11) followed by Egypt (9). For low‐fragility contexts, the greatest number of studies were from Ethiopia (26) followed by Bangladesh (12). Overall, the number of impact evaluations from conflict‐affected countries was low, and the same studies were included in many systematic reviews.

##### Funding agencies

**Figure 14:**
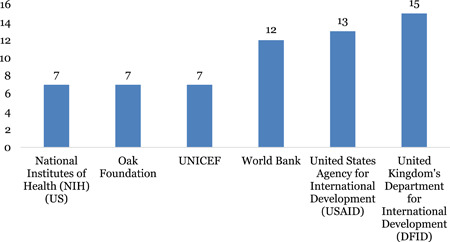
Top six funding agencies for VAC studies (impact evaluations and systematic reviews). VAC, violence against children

More than 90 funding agencies funded the 152 studies included in the EGM. The six agencies/organisations which supported the highest number of research and evaluation studies on VAC in LMICs were the United Kingdom Department for International Development (15 studies), followed by USAID, World Bank, UNICEF, Oak Foundation and National Institutes of Health. The top six funding agencies and the frequencies of studies supported by them are shown in Figure 14. The majority of the funding agencies were international and non‐profit organisations. The complete list of funding agencies is given in Supporting Information Appendix 9. Some studies were funded by more than one organisation/agency.

Education and life skills and income and economic strengthening are the top areas funded by both USAID and DFID. UNICEF supported seven studies out of the 152 studies included, in collaboration with other organisations or individually.

The UNICEF supported studies included in the present EGM are specifically from Bangladesh, Burkina Faso, Mexico, Nepal, South Africa, and Tanzania. In the included studies, UNICEF has supported the studies to assess the effectiveness of interventions to reduce child labour, child marriage, child abuse and maltreatment and intimate partner violence. UNICEF has supported numerous other intervention studies for reducing violence against women and children.

### 8.6 Synthesis of included studies by additional variables

#### 8.6.1 Publication of studies over time

**Figure 15:**
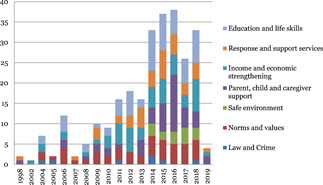
Trends in publication of studies on VAC by intervention. VAC, violence against children

There are 142 completed studies and 10 ongoing studies (which refer to registered protocols) included in the EGM. Figure 15 shows the number of systematic reviews and impact evaluation which evaluated the effects of interventions for reduction of VAC published each year between 2000 and 2019. The number of studies on VAC was low in late 1990s and early 2000s. In the year 2006, there was a spike in the number of studies, after which there was a gradual increase in the number of studies. The Millennium Development Goals came to an end with the launch of SDGs in 2015. The following year (2016) was the year when the highest number of studies to date was published (22 studies). Although there was no restriction of year on screening, the systematic reviews, included in the EGM, were conducted between the years 2009 and 2019.

**Figure 16:**
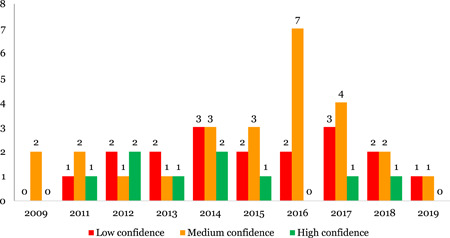
Confidence of systematic reviews over the years

The confidence of systematic reviews, as appraised for this EGM, remained consistent over the years with mostly medium‐confidence studies, followed by low‐confidence and then high‐confidence studies in number as shown in Figure 16.

#### 8.6.2 Ethical adequacy

An ethical adequacy tool was used for impact evaluation to establish the ethical considerations incorporated by the studies on a scale of high to low. Most of the RCTs followed strong ethical considerations (31) whereas the majority of the quasi‐experimental studies included low to moderate (23) ethical considerations.

**Figure 17:**
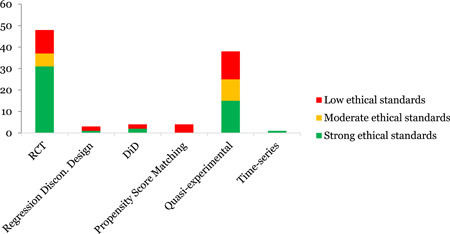
Ethical adequacy by study design among impact evaluations

The ethical considerations are plotted against study designs in Figure 17.

Our overall observation, however, is that there is a need to develop more tailored ethical adequacy appraisal tools better suited to the specific requirements of research on violence.

#### 8.6.3 Citations of included studies

Google Scholar citations were calculated for the 152 studies included and updated until 24 July 2019. The total citations were 9,585 and the average citations per study were 61.5 with a median of 19. The study Schultz P. (2004) had the highest number of citations (1,354 citations).

#### 8.6.4 Authors of the included Impact evaluations

There were 27 studies in which one author had more than one study in the area of VAC. Cluver L (three as first author and four as co‐author) has the highest number of studies in this area. Pulerwitz J has conducted three impact evaluations as first author and one as co‐author in the area of VAC. Gardner F, Heise L and Denison E have each conducted one study as first authors and three studies as co‐authors amongst the included studies. Peterman A has co‐authored three studies and Mikton, C has one study as a first author and three as a co‐author. Baird S, Diop NJ, Jewkes R, Parker B, Wendy K and Rosati FC are a few other authors who have conducted multiple studies in the area of VAC.

### 8.7 Risk of bias in included studies

#### 8.7.1 Systematic reviews

**Figure 18:**
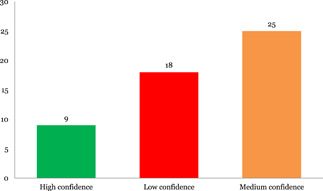
Number of systematic reviews by study confidence

Figure 18 shows the results of the critical appraisal of the 55 systematic reviews included in the map. Only 17% of the 55 systematic reviews included are high‐confidence systematic reviews, which means that more than three quarters (83%) are either low or medium confidence systematic reviews (18 and 25, respectively). This clearly suggested a dearth of high‐confidence systematic reviews in the area of interventions to address VAC. There were three (3) ongoing reviews.

The reason for a high number of low‐confidence systematic reviews lies in factors such as reviews have been produced by organisations or individuals that have not complied with international checklists and standards which are readily available for production of systematic reviews. Also, the search and screening procedures were not as per standard guidelines, and studies were insufficiently transparent with respect to methodology and did not provide the list and reasons for excluded studies.

#### 8.7.2 Impact evaluations

**Figure 19:**
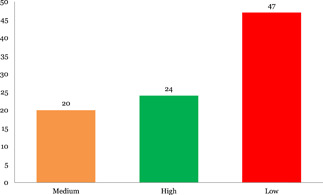
Number of impact evaluations by study confidence

Figure 19 shows the results of the critical appraisal of the included 97 impact evaluations. Study confidence was rated high, medium or low for each of the criteria, applying the standards as stated in the critical appraisal tool given in the report (given in Supporting Information Appendix 7).

Only 30% of the 97 included impact evaluations were high confidence, which means that 70% were either low of medium confidence (47 and 20, respectively). The large number of low‐confidence studies is largely driven by concerns related to attrition. Sixty‐six percent (66% of the impact evaluations) had no mention of power calculation. Attrition was not reported in nearly half (40) of the included impact evaluations and baseline balance was not reported for 32 studies. Study designs also played a role in determining the confidence of studies. Before and after studies scored low according to the criteria of the critical appraisal tool.

## 9 DISCUSSION AND GAPS IN EVIDENCE

### 9.1 Summary of main results

We summarise some of the emerging findings and lessons in this section. There is a clear need for more and better confidence impact evaluations across all INSPIRE categories and better use of systematic reviews and implementation research to complement more rigorous evaluations across many components of INSPIRE intervention categories. Geographical coverage remains skewed to a few regions and a few countries within them.

### 9.2 Areas of evidence concentration

Most of the impact evaluations measured impact of “education and life skills” and “income and economic strengthening” in reducing all forms of VAC. The intervention category “Parent, child and caregiver support” had a fair concentration of studies as well.

In terms of outcomes, impacts on “changing norms and values” and “health” impacts were the most studied outcomes apart from direct reduction of VAC.

The distribution of studies (systematic reviews and impact evaluations) across regions is uneven. As regions, Sub‐Saharan Africa and South Asia had the highest number of studies. Country‐wise South Africa, India, Uganda and Ethiopia have the largest number of studies.

### 9.3 Areas of major gaps in the evidence, and confidence considerations

Many areas of the map are sparsely populated and even the areas with existing evidence are generally of low confidence. There are pockets of high‐confidence evidence, but there is an overall low number of studies from LMICs in the area of VAC. Evidence gaps are noted in the areas of “law, crime and justice” and “safe environments”.

More detailed analysis of intervention‐outcome‐specific analysis reveal that even in high areas of evidence, there are pockets of evidence gaps. For example, though income and economic strengthening is fairly covered, there are gaps in evidence on insurance and welfare schemes and income generation and credit interventions. Similar gaps are seen in parent, child and caregiver support, where there are lack of studies on maternal/paternal mental health and peer‐relationship and training interventions.

An emerging insight from our EGM is that the evidence seems to be concentrated on reducing victimisation and there seem to be gaps in evidence on the effectiveness of interventions for perpetration of violence.

There seems to be differences in understanding and defining diverse forms of VAC, as our findings show that a large proportion of the studies reported interventions for “unclassified” types and forms of violence.

A significant share of the systematic review evidence base has methodological limitations. These limitations include unclear inclusion criteria for interventions and topics, no use of independent screening or data extraction, missing risk of bias assessments of included studies and use of vote‐counting approaches.

A significant share of impact evaluations also had methodological limitations. These limitations include high attrition rates, unclear interventions and outcome definition and lack of power calculations in the sample.

It was rare for studies to include information on cost‐analysis (including cost‐effectiveness and cost‐benefit analysis). There were only two studies that conducted cost‐analysis.

Evidence is scarce on assessing the effectiveness of “maternal and paternal mental health”, “children in care” and “media and communication” interventions. Only five studies noted the effectiveness of “insurance and welfare schemes” interventions in reduction of VAC and they are noted to be of medium to high confidence.

There are also evidence gaps in studies assessing the effectiveness of interventions for reduction of VAC of less than 3 years of age and of children belonging to ethnic minorities or children with disabilities.

There are major gaps in studies assessing the effectiveness of parent–child and caregiver support on safety and risk factors for other harms and economic and social outcomes. Evidence was lacking in terms of the impact of the intervention area “response and support services” on economic and social outcomes.

There is limited evidence on the studies assessing effectiveness of interventions related to specific forms of violence such as intimate partner violence amongst children and adolescent, peer violence and corporal punishment.

There are very few studies from low‐income countries as compared with middle‐income countries (low and upper‐middle). This is of concern because many low‐income countries from the regions report high violence prevalence against children and the need for effective interventions is critical.

The EGM analysis showed that there are many studies from Sub‐Saharan Africa, but it is mainly concentrated in a few countries, particularly South Africa. Many LMICs from Sub‐Saharan Africa have minimal or no evidence (at least in English language publications). Gaps from Francophone Sub‐Saharan Africa and other regions such as Latin America and the Caribbean, Middle East and North Africa may be addressed in Phase 2 when evidence published in five other languages—Arabic, Chinese, French, Portuguese and Spanish—are included in the EGM.

### 9.4 Limitations of the EGM

Violence is as complex issue, with a range of intersecting forms, drivers and risk factors operating at the level of the child, family, community and wider institutions. This EGM has been based on an as expansive a framework as possible, though limitations may remain in the scope and approach. We trust and expect that other researchers will address limitations going forward, contributing to more robust evidence architecture to inform this field.

Other limitations:

1. This search was limited to English language only and hence we may have missed potentially relevant studies in other languages. This will be corrected in a second phase of work that will update the EGM with literature from five languages—Arabic, Chinese, French, Portuguese and Spanish in 2020/2021.
2. Searching the “grey” literature is challenging and there are possibilities of missing a few studies. However, to strengthen the search, we included limited grey literature on the advice of the advisory group.
3. Purely qualitative studies were not included in the EGM, thus presenting a limitation that needs attention in future exercises or updates. We included mixed‐method studies.
4. Hand searching was not done for the current EGM, which limits the search to articles only available online.
5. Cyber/online was not included as a search term, though all forms of violence and bullying were searched for.

### 9.5 Recommendations

Main recommendations from the findings of the EGM:

1. The EGM should be the basis to identify gaps in evidence such as on key intervention areas and forms of violence, so that investments in research can be better targeted to address gaps.
2. The EGM finds that there is a need to improve the confidence of impact evaluations. This includes standardising definitions of violence used, study design, implementation and also reporting.
3. Organisations funding research need to direct more funding and give strategic priority to confidence impact evaluations, mixed‐method studies and systematic reviews, designed to improve and strengthen methodology, and require their grantees to conduct long‐term follow‐up surveys and cost‐analyses.
4. Researchers should increase measurement of changes in gendered social norms as an outcome; and include vulnerable populations, such as children with disabilities, children in institutions, children who are street‐connected, and children from low‐income group and ethnic minorities, in the sample.
5. The requirement to report on the application of ethical standards must be made mandatory as part of funding applications, as well as research publication. There is a need for a specific, tailored tool for ethical appraisal of research on violence, and ethical considerations need to be included in confidence appraisals as well.
6. The EGM should be made a living review and expanded to include qualitative literature, to provide a database for researchers to update with relevant literature, as well as for policy makers and other stakeholders to have easily accessible summaries of relevant literature for their interest.
7. More high‐confidence impact evaluations should be funded and implemented. One way to ensure confidence can be by adhering to the standardised international checklists for relevant study design (available for impact evaluations as well as for systematic reviews) and making training available and adaptable to context.
8. In the areas of high evidence concentration such as education and life skills and income and economic strengthening, evidence summaries should be generated gain an idea of the extent of the developing country literature, and also to develop taxonomy of approaches relevant in these contexts.
9. As a next step, this EGM can provide a preliminary content for the web based evidence portal for VAC in LMICs. Wider stakeholder consultations with experts in the field is needed.

### 9.6 Stakeholder engagement throughout the EGM process

1. A consultative process was followed for refining the EGM content including through the establishment of an expert advisory group. An early presentation of the approach was made in March 2019 at a meeting held at UNICEF Office of Research–Innocenti. Further presentations were made in the What Works Global Summit (WWGS), Mexico, 16–18 October 2019 and in the 19th European Conference on Developmental Psychology, Athens, August 29–September 1 2019 to table emerging findings from the map.
2. The advisory group reflected and commented on the technical and content aspects of the title registration and protocol for the EGM. The advisory group shared feedback on EGM report in August 2019 and editing was done accordingly. The final report was reviewed by most members of the Advisory group over June and July 2020. The authors responsible for primary contact with the advisory group were RS, HW and AS. The details of the advisory group are given on page 29–30 of the present report. Final responsibility for content rests with the authors.
3. A peer review process was followed for the publication of protocol. The peer reviewers were experts in the field of VAC and systematic review methodology. The Campbell Social Welfare Coordinating group shared editor's comments on the protocol. In addition to the advisory group and peer reviewers, comments on the report were solicited within UNICEF and the Global Partnership to End Violence against Children.

## 10 AUTHORS’ CONCLUSIONS

### 10.1 Implications for research, practice and/or policy

This EGM provides a valuable snapshot of the available evidence on the effectiveness of interventions for the VAC, but it does not tell us what that evidence says. The maps are a first building block to construct an evidence architecture for the sector. There is considerable need for more research to widen and deepen understanding on what works to reduce VAC in different settings.

➣ A more standardised approach (adaptable to context) is required to generate evidence on the effectiveness of these interventions in reducing different types and forms of violence. Currently, there seem to be overlaps between different forms of violence without clear delineation in many studies. Researchers must address effectiveness of interventions for specific forms of violence in different contexts to have a better understanding on what works, where, why and for whom.
➣ More studies are needed to assess the effectiveness of interventions for perpetration of VAC within the INSPIRE interventions framework.
➣ More studies are needed from low‐income and conflict‐affected settings.
➣ More high‐confidence systematic reviews and impact evaluations need to be commissioned with standardised guidelines and tailored guidance to ensure robustness, as most of them included in the map were found to be of low confidence.
➣ More impact evaluations are needed that report outcomes for vulnerable groups of children, requiring larger sample sizes in studies and better disaggregation in study design, methodology and reporting.
➣ More impact evaluations are needed that assess gendered effects of interventions and on diverse social groups in a given context, utilising mixed methods to better understand for whom the interventions work, and how.
➣ Ethical standards need to be better‐defined and made a mandatory component of all research studies. UNICEF has set global standards for UNICEF staff and partners conducting ethical research involving children https://www.unicef-irc.org/research/ethical-research-and-children/. These standards have set global benchmarks and a detailed database is available at www.childethics.com. However, there is an urgent need for a more standardised ethical tool to assess these studies.
➣ Violence is a complex issue, with a range of intersecting forms, drivers and risk factors operating at the level of the child, family, community and wider institutions. Entry‐points to prevent violence are several, as the INSPIRE framework elaborates. To sharpen the design of programmes and interventions across sectors, there is a need to increase investment in evidence‐informed policy and programmatic learning and advocacy.

## AUTHOR CONTRIBUTIONS

Content: Ramya Subrahmanian has an extensive experience in research, policy advocacy, training and teaching. She has experience in use of evidence across all of UNICEF's policy areas. In her previous capacity as Executive Director, Know Violence in Childhood, she oversaw the commissioning of over 45 new papers on violence prevention including systematic reviews on LMICs, as well as the publication of an updated annotated bibliography.

EGM methods: Ashrita Saran and Howard White have previous experience in systematic review methodology, including searching, data collection, and theory‐based synthesis, which means they are proficient in carrying out the various processes in an EGM, such as search, eligibility screening, confidence assessment and coding. They have undertaken an overview of approaches to mapping in a range of organisations. Jill Adona is an experienced screener and coder who has previously worked on Campbell Collaboration research projects. Jill has attended training workshops on evidence synthesis provided by both 3ie and Campbell. Prachi Pundir has experience in systematic reviews and has previously worked on systematic reviews and meta‐analysis with Public Health Evidence South Asia, Manipal, and all authors are proficient in carrying out the various processes in an EGM, such as eligibility screening, confidence assessment and coding.

Statistical analysis: Ashrita Saran and Prachi Pundir have training in data management and using statistical softwares for quantitative data analysis.

Information retrieval: Ashrita Saran and Prachi Pundir have training in designing and implementing search strategies.

P. P, A. S., H. W., J. A. and R. S. contributed to writing and revising this report. The search strategy was developed, validated and piloted by P. P. and A. S. Search was conducted by P. P., A. S. and J. A. P. P. will be responsible for updating this EGM.

## CONFLICT OF INTERESTS

One of the co‐authors is an Innocenti staff member who commissioned the study, helped shape the search strategy, and worked with the Campbell Collaboration team to coordinate internal and external review processes and to contextualise findings and recommendations. The main search, screening, coding and analysis were independently conducted by Campbell Collaboration staff independently.

## PLANS FOR UPDATING THE EGM

Once completed, the EGM is planned to be updated yearly, provided we have availability of funds. The lead author and/or the corresponding authors will be responsible for updating the EGM.

## DIFFERENCES BETWEEN PROTOCOL AND MAP

In the course of finalising this map, there have been some deviations from the protocol, informed by practical considerations:

1. “Intimate partner violence”, “peer violence” and “corporal punishment” was added as forms of violence.
2. A typology of violence used in the protocol on “community violence and collective violence” was removed as it was found to be beyond the scope of this map. The focus for this map was to capture studies on interpersonal VAC.
3. We added “perpetration of violence” as a filter.

## SOURCES OF SUPPORT

Financial support for this research was provided by UNICEF Office of Research—Innocenti. Non‐financial support was not received for the production of this map.

## Supporting information

Supporting information
