# Supplementary material for: Interventions for reducing violence against children in low‐ and middle‐income countries: An evidence and gap map
Source: Campbell Syst Rev. 2020 Oct 20;16(4):e1120. doi: 10.1002/cl2.1120 (PMC8356324; doi:10.1002/cl2.1120)

**Appendix**

**Appendix 1: Definitions**

Law, crime and justice:

A) Laws: The system of rules which a particular country or community recognizes as regulating the actions of its members and which it may enforce by the imposition of penalties.

B) Crime and justice system: The system of law enforcement that is directly involved in apprehending, prosecuting, defending, sentencing, and punishing those who are suspected or convicted of criminal offenses.

Norms and values: Aims to alter the social expectations that define “appropriate” behaviour for women and men, such as norms that dictate men have the right to control women, and which make women and girls vulnerable to physical, emotional and sexual violence by men.

A) Community mobilization programmes: Community mobilization engages all sectors of the population in a community wide effort to address a health, social, or environmental issue. It brings together policy makers and opinion leaders, local, state, and federal governments, professional groups, religious groups, businesses, and individual community members. Community mobilization empowers individuals and groups to take some kind of action to facilitate change.

B) Bystander interventions: Bystander Intervention is a social science model that predicts the likelihood of individuals (or groups) willing to actively address a situation they deem problematic. A bystander is anyone who observes any situation.

Safe environments:

A) Child safe environments: Child safe environments are safe and friendly settings where children feel respected, valued and encouraged to reach their full potential.

B) School WASH & Infrastructure: WASH is the collective term for Water, Sanitation and Hygiene. Due to their interdependent nature, these three core issues are grouped together to represent a growing sector. While each a separate field of work, each is dependent on the presence of the other. Buildings, classrooms, laboratories, and equipment are education infrastructure.

Parent, child and caregiver support:

A) Parent-training and education: interventions that promote positive parenting practice

B) Parental mental health: Mental health is defined as a state of well-being in which every individual realizes his or her own potential, can cope with the normal stresses of life, can work productively and fruitfully, and is able to make a contribution to her or his community. Maternal and paternal refers to the affected child/children’s mother or father respectively.

C) Peer/relationship training: Peer educators are typically the same age or slightly older than the group with whom they are working. Peer education is based on the reality that many people make changes not only based on what they know, but on the opinions and actions of their close, trusted peers. Peer educators can communicate and understand in a way that the best-intentioned adults can't and can serve as role models for change.

1. Economic transfers: Social assistance help the poor and vulnerable cope with crises and shocks, find jobs, invest in the health and education of their children, and protect the aging population. It includes cash transfers, public works, in-kind transfers, subsidies or school feeding.

Cash transfers can broadly be categorised into two types: Conditional and Unconditional cash transfers. Conditional cash transfer programmes give money to households on the condition that they comply with certain pre-defined requirements. These conditions can include, for example, up-to-date vaccinations, regular visits to a health care facility, regular school attendance by children, and complying with health and nutrition promotion activities (e.g. attending education sessions, taking nutritional supplements, etc.). Conditional cash transfer programmes are aimed at reducing poverty as well as breaking the cycle of poverty for the next generation through the development of human capital. Unconditional cash transfers are similar in nature, however, they do not include co-responsibilities for receiving benefits. One popular form of unconditional transfers includes universal basic income interventions, where every citizen receives an unconditional basic income (however these are rare in practice, particularly in LMICs).

B) Income generating or savings/credit interventions: Income Generation interventions attempt to address poverty, unemployment, and lack of economic opportunities to increase participants’ ability to generate income and secure livelihoods.

C) Broad-based Social Protection (including insurance and welfare schemes): Social protection represents a large range of policies and programming including employer and labour force benefits (including unemployment befits, maternity leave policies), pensions and retirement benefits, disability benefits and other labor market programs, social insurance and other social welfare services. Social insurance is comprised of programs that minimize the negative impact of economic shocks on individuals and families. They include publicly provided or mandated insurance schemes against disability and death of the main household provider, and health insurance. Social protection can be both contributory or non-contributory.

Response and support services:

A) Counselling and therapeutic approaches: Counselling is a learning-oriented process, which occurs usually in an interactive relationship, with the aim of helping a person learn more about the self, and to use such understanding to enable the person to become an effective member of society.

B) Screening and training: Screening and reporting combined with interventions. Training the health professional/social workers/teachers for violence against children.

C) Creating safe spaces: Child friendly spaces can be defined as places designed and operated in a participatory manner, where children affected by natural disasters or armed conflict can be provided with a safe environment, where integrated programming including play, recreation, education, health, and psychosocial support can be delivered and/or information about services/supports provided.

D) Media and communication: Communication encompasses several areas including health journalism, edutainment, education, interpersonal communication, media advocacy, organizational communication, risk and crisis communication, social communication and social marketing.

Education and life skills:

A) Sexual and reproductive health education: Good sexual and reproductive health is a state of complete physical, mental and social well-being in all matters relating to the reproductive system. To maintain one’s sexual and reproductive health, people need access to accurate information and the safe, effective, affordable and acceptable contraception method of their choice.

B) Life and social skills training: are designed to help children and adolescents manage anger, resolve conflict and develop the necessary social skills to solve interpersonal problems without violence, and are usually implemented in school settings.

**Appendix 2: Search query (Medline)**

We prepared a search strategy using the title, abstract (text word) terms together with each database’s indexing terminology, where available.

**1. Developing Country Keywords:**

((developing countr* or developing nation* or developing population* or "developing world" or less developed countr* or less developed nation* or less developed population* or "less developed world" or lesser developed countr* or lesser developed nation* or lesser developed population* or "lesser developed world" or under developed countr* or under developed nation* or under developed population* or "under developed world" or underdeveloped nation* or underdeveloped population* or "underdeveloped world" or middle income countr* or middle income nation* or middle income population* or low income countr* or low income nation* or low income population* or lower income countr* or lower income nation* or lower income population* or underserved countr* or underserved nation* or underserved population* or "underserved world" or under served countr* or under served nation* or under served population* or "under served world" or deprived countr* or deprived nation* or deprived population* or "deprived world" or poor countr* or poor nation* or poorer countr* or poorer nation* or poorer population* or developing economy* or less developed econom* or lesser developed econom* or under developed econom* or underdeveloped econom* or middle income econom* or low income econom* or lower income econom* or "low gdp" or "low gnp" or "low gross domestic" or "lower gdp" or "lower gnp" or "lower gross domestic" or "lower gross national" or lmic or lmics or "third world" or lami countr* or transitional countr* or L&MIC or LAMIC or LDC).mp. or LIC.ti,ab,kw.

(Africa OR Algeria OR Angola OR Benin OR Botswana OR Burkina Faso OR Burundi OR Cameroon OR "Cape Verde" OR "Central African Republic" OR Chad OR "Democratic Republic of the Congo" OR "Republic of the Congo" OR Congo OR "Cote d'Ivoire" OR "Ivory Coast" OR Djibouti OR Egypt OR "Equatorial Guinea" OR Eritrea OR Ethiopia OR Gabon OR Gambia OR Ghana OR Guinea OR Guinea-Bissau OR Kenya OR Lesotho OR Liberia OR Libya OR Madagascar OR Malawi OR Mali OR Mauritania OR Morocco OR Mozambique OR Namibia OR Niger OR Nigeria OR Rwanda OR "Sao Tome" OR Principe OR Senegal OR "Sierra Leone" OR Somalia OR Somaliland OR "South Africa" OR "South Sudan" OR Sudan OR Swaziland OR Tanzania OR Togo OR Tunisia OR Uganda OR Zambia OR Zimbabwe).ti,ab,kw.

("South America" OR "Latin America" OR "Central America" OR Mexico OR Argentina OR Bolivia OR Brazil OR Chile OR Colombia OR Ecuador OR Guyana OR Paraguay OR Peru OR Suriname OR Uruguay OR Venezuela OR Belize OR "Costa Rica" OR "El Salvador" OR Guatemala OR Honduras OR Nicaragua OR Panama).ti,ab,kw.

("Middle East" OR "South-East Asia" OR "Indian Ocean Island*" OR "South Asia" OR "Central Asia" OR Caucasus OR Afghanistan OR Azerbaijan OR Bangladesh OR Bhutan OR Burma OR Cambodia OR China OR Georgia OR India OR Iran OR Iraq OR Jordan OR Kazakhstan OR Korea OR "Kyrgyz Republic" OR Kyrgyzstan OR Lao OR Laos OR Lebanon OR Macao OR Mongolia OR Myanmar OR Nepal OR Oman OR Pakistan OR Russia OR "Russian Federation" OR "Saudi Arabia" OR Bahrain OR Indonesia OR Malaysia OR Philippines OR Sri Lanka OR Syria OR "Syrian Arab Republic" OR Tajikistan OR Thailand OR Timor-Leste OR Timor OR Turkey OR Turkmenistan OR Uzbekistan OR Vietnam OR "West Bank" OR Gaza OR Yemen OR Comoros OR Maldives OR Mauritius OR Seychelles).ti,ab,kw.

("Pacific Islands" OR "American Samoa" OR Fiji OR Guam OR Kiribati OR "Marshall Islands" OR Micronesia OR New Caledonia OR "Northern Mariana Islands" OR Palau OR "Papua New Guinea" OR Samoa OR "Solomon Islands" OR Tonga OR Tuvalu OR Vanuatu).ti,ab,kw

("Eastern Europe" OR Balkans OR Albania OR Armenia OR Belarus OR Bosnia OR Herzegovina OR Bulgaria OR Croatia OR Cyprus OR "Czech Republic" OR Estonia OR OR Kosovo OR Latvia OR Lithuania OR Macedonia OR Malta OR Moldova OR Montenegro OR OR Romania OR Serbia OR "Slovak Republic" OR Slovakia OR Slovenia OR Ukraine).ti,ab,kw.

(Afghanistan OR Albania OR Algeria OR American Samoa OR Angola OR Armenia OR Azerbaijan OR Bangladesh OR Belarus OR Belize OR Benin OR Bhutan OR Bolivia OR Bosnia and Herzegovina OR Botswana OR Brazil OR Bulgaria OR Burkina Faso OR Burundi OR Cabo Verde OR Cambodia OR Cameroon OR Central African Republic OR Chad OR China OR Colombia OR Comoros OR Congo, Dem. Rep. OR Congo, Rep. OR Costa Rica OR Côte d'Ivoire OR Cuba OR Djibouti OR Dominica OR Dominican Republic OR Ecuador OR Egypt, Arab Rep. OR El Salvador OR Equatorial Guinea OR Eritrea OR Ethiopia OR Fiji OR Gabon OR Gambia, The OR Georgia OR Ghana OR Grenada OR Guatemala OR Guinea OR Guinea-Bissau OR Guyana OR Haiti OR Honduras OR India OR Indonesia OR Iran, Islamic Rep. OR Iraq OR Jamaica OR Jordan OR Kazakhstan OR Kenya OR Kiribati OR Korea, Dem. People's Rep. OR Kosovo OR Kyrgyz Republic OR Lao PDR OR Lebanon OR Lesotho OR Liberia OR Libya OR Macedonia, FYR OR Madagascar OR Malawi OR Malaysia OR Maldives OR Mali OR Marshall Islands OR Mauritania OR Mauritius OR Mexico OR Micronesia, Fed. Sts. OR Moldova OR Mongolia OR Montenegro OR Morocco OR Mozambique OR Myanmar OR Namibia OR Nauru OR Nepal OR Nicaragua OR Niger OR Nigeria OR Pakistan OR Papua New Guinea OR Paraguay OR Peru OR Philippines OR Romania OR Russian Federation OR Rwanda OR Samoa OR São Tomé and Principe OR Senegal OR Serbia OR Sierra Leone OR Solomon Islands OR Somalia OR South Africa OR South Sudan OR Sri Lanka OR St. Lucia OR St. Vincent and the Grenadines OR Sudan OR Suriname OR Swaziland OR Syrian Arab Republic OR Tajikistan OR Tanzania OR Thailand OR Timor-Leste OR Togo OR Tonga OR Tunisia OR Turkey OR Turkmenistan OR Tuvalu OR Uganda OR Ukraine OR Uzbekistan OR Vanuatu OR Venezuela, RB OR Vietnam OR West Bank and Gaza OR Yemen, Rep. OR Zambia OR Zimbabwe).mp.

**2. Population Keywords:**

(child* OR young child* OR pre-schooler* OR kindergarten* OR early child or childhood or early year*).ti,ab,kw.

(juvenile* OR minors or youth OR "young adult* " OR "young wom$n" OR "young m$n" OR girl* OR boy* OR (school adj6 student*) OR teen* OR schoolgirl* OR schoolboy*).ti,ab,kw

(pupil* OR student* OR partner* OR spouse* OR peer* OR romantic partner* OR boy friend* OR girlfriend* or girl friend* OR acquaintance* OR non stranger* OR nonstranger*).ti,ab,kw.

(adolescen* OR boy$1 OR boyhood OR girl* OR teen* OR preteen* OR pubescen* OR prepubescen* or youth* OR juvenile* OR preteen* OR pre teen* OR young people* OR young person* OR early adult* OR young adult* OR infan* OR baby or babies OR neonate* OR newborn* ).ti,ab,kw.

**3. Violence Keywords:**

(Violen* or Aggress*) NOT ("cancer* OR disease* OR neoplasm* ").ti,ab,kw.

(abus* OR maltreat* OR mistreat* OR ill-treat* OR harm* OR victimi* OR ill-use* OR misuse* ORmis-use* OR oppress* OR violat* OR batter* OR bully OR bullied).ti,ab,kw.

(Sexual* adj5 (assault or harassment or exploitation or traffic* or slave*)).ti,ab,kw.

(human traffic* OR harmful traditional practice* OR female genital mutilation OR FGM OR female genital cutting OR slavery OR forced prostitution OR forced marriage* OR early marriage* ).ti,ab,kw.

(harsh parent* OR corporal punishment OR beat* OR physical discipline or paddling OR spank* OR bully* OR fight* OR fought OR assault* ).ti,ab,kw.

**4. Study design Keywords:**

(systematic* OR synthes*) adj3 (research OR evaluation* OR finding* OR thematic* OR report OR descriptive OR explanatory OR narrative OR meta* OR review* OR (map adj3 (evidence or gap)).ti,ab,kw.

("meta regression" OR "meta synth*" OR "meta-synth*" OR "meta analy*" OR "metaanaly*" OR "meta-analy*" OR "metanaly*" OR "metaregression" OR "metaregression" OR "methodologic* overview" OR "pool* analys*" OR "pool* data" OR "quantitative* overview" OR "research integration").ti,ab,kw.

( "impact evaluation" OR counterfactual OR experiment* OR "quasi-experimental" OR “discontinuity design” OR "discontinuity regression" OR " regression discontinuity" OR “fixed effect*” OR regression OR “difference* in difference*” OR “double differenc*” OR “instrumental variable*” OR "propensity score matching" OR matching OR “propensity weight*” OR “interrupted time-series” OR "panel data" OR "double robust" OR “random* control*” OR randomi?ation OR "random* trial*" OR "control group" OR “impact assessment” OR “econometric analys*” OR “cross-sectional data” OR “difference-in-difference).ti,ab.kw.

(cross sectional OR observational OR retrospective OR longitudinal OR survey* OR cohort OR follow up OR follow up OR case-control OR case control OR mixed method OR mixed-method).ti,ab.sh.

**Example of search in Medline:**

| **Results** | **Type** | **Actions** |
| --- | --- | --- |
| 1 | (Violen* or Aggress*).mp. not "cancer* OR disease* OR neoplasm* ".ti,ab,kw. [mp=title, abstract, full text, caption text] | 389984 |
| 2 | (abus* or maltreat* or mistreat* or ill-treat* or harm* or victimi* or ill-use* or misuse* or mis-use* or oppress* or violat* or batter* or bully or bullied).ti,ab,kw. | 79845 |
| 3 | (Sexual* adj5 (assault or harassment or exploitation or traffic* or slave*)).ti,ab,kw. | 1762 |
| 4 | (human traffic* or harmful traditional practice* or female genital mutilation or FGM or female genital cutting or slavery or forced prostitution or forced marriage* or early marriage*).ti,ab,kw. | 645 |
| 5 | (harsh parent* or corporal punishment or beat* or physical discipline or paddling or spank* or bully* or fight* or fought or assault*).ti,ab,kw. | 21755 |
| 6 | ((systematic* or synthes*) adj3 (research or evaluation* or finding* or thematic* or report or descriptive or explanatory or narrative or meta* or review*)).mp. or (map adj3 (evidence or gap)).ti,ab,kw. [mp=title, abstract, full text, caption text] | 257933 |
| 7 | ("meta regression" or "meta synth*" or "meta-synth*" or "meta analy*" or "metaanaly*" or "meta-analy*" or "metanaly*" or "metaregression" or "metaregression" or "methodologic* overview" or "pool* analys*" or "pool* data" or "quantitative* overview" or "research integration").ti,ab,kw. | 53728 |
| 8 | (random$ or placebo$ or single blind$ or double blind$ or triple blind$).ti,ab. | 292426 |
| 9 | (systematic$ adj2 (review$ or overview)).ti,ab. | 54334 |
| 10 | (quantitativ$ adj5 synthesis$).tw. | 8719 |
| 11 | (quantitativ$ adj5 review$).tw. | 11224 |
| 12 | (cross sectional or observational or retrospective or longitudinal or survey* or cohort or follow up or follow up or case-control or case control or mixed method or mixed-method).ti,ab,kw. | 691354 |
| 13 | (developing countr* or developing nation* or developing population* or "developing world" or less developed countr* or less developed nation* or less developed population* or "less developed world" or lesser developed countr* or lesser developed nation* or lesser developed population* or "lesser developed world" or under developed countr* or under developed nation* or under developed population* or "under developed world" or underdeveloped nation* or underdeveloped population* or "underdeveloped world" or middle income countr* or middle income nation* or middle income population* or low income countr* or low income nation* or low income population* or lower income countr* or lower income nation* or lower income population* or underserved countr* or underserved nation* or underserved population* or "underserved world" or under served countr* or under served nation* or under served population* or "under served world" or deprived countr* or deprived nation* or deprived population* or "deprived world" or poor countr* or poor nation* or poorer countr* or poorer nation* or poorer population* or developing economy* or less developed econom* or lesser developed econom* or under developed econom* or underdeveloped econom* or middle income econom* or low income econom* or lower income econom* or "low gdp" or "low gnp" or "low gross domestic" or "lower gdp" or "lower gnp" or "lower gross domestic" or "lower gross national" or lmic or lmics or "third world" or lami countr* or transitional countr* or L&MIC or LAMIC or LDC).mp. or LIC.ti,ab,kw. [mp=title, abstract, full text, caption text] | 185155 |
| 14 | (child* or young child* or pre-schooler* or kindergarten* or early child or childhood or early year*).ti,ab,kw. | 336982 |
| 15 | (juvenile* or minors or youth or "young adult* " or "young wom$n" or "young m$n" or girl* or boy* or (school adj6 student*) or teen* or schoolgirl* or schoolboy*).ti,ab,kw. | 105160 |
| 16 | (pupil* or student* or partner* or spouse* or peer* or romantic partner* or boy friend* or girlfriend* or girl friend* or acquaintance* or non stranger*).mp. or nonstranger*.ti,ab,kw. [mp=title, abstract, full text, caption text] | 1124674 |
| 17 | (adolescen* or boy$1 or boyhood or girl* or teen* or preteen* or pubescen* or prepubescen* or youth* or juvenile* or preteen* or pre teen* or young people* or young person* or early adult* or young adult* or infan* or baby or babies or neonate* or newborn*).ti,ab,kw. | 286117 |
| 18 | 1 or 2 or 3 or 4 or 5 | 471292 |
| 19 | 6 or 7 or 8 or 9 or 10 or 11 or 12 | 1120191 |
| 20 | 18 and 19 | 130953 |
| 21 | 13 and 20 | 9245 |
| 22 | 14 or 15 or 16 or 17 | 1524337 |
| **23** | **21 and 22** | **5776** |

**Appendix 3: Detailed Eligibility criteria**

|  | **Include** | **Exclude** |
| --- | --- | --- |
| Literature type | Published journal articles  Grey literature including technical reports which report quantitative data related to effectiveness of interventions for violence against children  Technical reports/ working papers | Commentary or conceptual papers  Editorial  Conference proceedings  Case studies |
| Study design | All study designs must include quantitative data  Permissible study designs include:  Systematic reviews  Metanalysis/ meta regressions  Randomised controlled trial  Modelling with empirically grounded parameters/ Econometric studies (Regression discontinuity, Propensity score or other matching techniques, Difference in difference)  Instrumental variables  Other matching designs  Rigorous quasi-experimental design/ Quasi-experimental  Natural experiments  Single-subject design  Analytical observational  Before-after studies  Time-series | Purely qualitative studies were excluded regardless of design.  Descriptive studies (cross-sectional studies) reporting data narratively, but do not give statistical analysis  Literature reviews |
| Population | Children in the group of less than or equal to 18 years  Age group is classified based on the WHO age criteria stated as follows: Infanthood (<3 years of age), Childhood (3-10 years), Adolescence (10-18 years).  Children from Low- and Middle-Income Countries as defined by World Bank classification.  We have included studies that have age-group including both children and adults provided the interventions are directed towards children (having age range 0-18 years, but have excluded studies with beneficiary population above 18 years).  For systematic reviews with global focus, we:  Excluded the Systematic reviews that include studies only from High income even if they did not have any search restrictions.  Population sub-group of interest included: Orphans, children with disabilities, children belonging to ethnic minorities, child sex workers, child brides, isolated children/street children, children with HIV/AIDS and children in conflict and humanitarian settings. | Studies with target age group or beneficiaries (indirect) above 18 years of age. |
| Interventions | We included studies with interventions that aim reduce violence against children as a primary focus.  These interventions were based on INSPIRE guidelines and include categories as:  1. Laws, crime and justice.  2. Norms and values: Community mobilization programmes, Bystander interventions, Media campaigns including mass media and education.  3. Safe environments: Making existing environments safe (through design changes), Creating safe places, hotspot evaluation approaches  4. Parent, child and caregiver support: Parent-training and education - interventions that promote positive parenting practices, Maternal/Paternal mental health, Peer/ relationship training, Parent and child support groups, Government agencies that coordinate/ streamline all activities related to parenting and parent support.  5. Income and economic strengthening: Broad-based social protection (Economic transfers), Income generating or savings/ credit interventions, Insurance and welfare schemes  6. Response and support services: Counselling and therapeutic approaches, Screening and training, Children in care, Media and communication  7. Education and life skills: Gender transformative approaches, Life and social skills training | Interventions that focus on outcome related to child neglect, negligent behavior were excluded  Interventions not focused on children |
| Outcomes | We included studies that aim to reduce violence against children as a primary outcome and including following:  1. Violence: Sexual violence, Physical violence, Emotional/psychological violence (Financial abuse)  2. Norms, values, belief and attitude: Belief on parenting practices, Gender roles, Delinquent, violent and other risk-taking behavior (including reoffending, recidivism rates), Empowerment  3. Health: Substance abuse, Child development and child mental health, Maternal mental health, Morbidity and mortality, Sexual and reproductive health  4. Safety and risk factors for other harms: Social isolation (homeless and street connected children), Female Genital Mutilation (FGM) and child marriage, Child labour/ trafficking, Safe environment/spaces  5. Economic and social: Poverty and food security, Employment and labour force participation, Savings and credit, Social discrimination (caste, race, ethnicity), social inclusion and gender equity  6. Cost Analysis: Cost-effectiveness, Cost-benefit  Education  7. School enrolment & attendance: School performance, WASH & Infrastructure, Gender roles and life skills |  |

**Appendix 4: Screening tool**

Is there an intervention for violence in the study (physical/ mental/ sexual abuse/ injury/ maltreatment or exploitation)?

**NO**

**YES**

Does the study report quantitative data?

Is the study outcome stated as child neglect (Emotional/ physical/ educational)?

Is the target age-group for intervention or beneficiaries (indirect) 0-18 years/ including 18 years age-group?

**YES**

**NO**

EXCLUDE

**YES**

EXCLUDE

Include in the EGM analysis

**NO**

EXCLUDE

**NO**

Does the study provide any other statistical data to prove effectiveness?

**NO**

**YES**

Is the study an impact evaluation or systematic review with violence as a primary/ secondary outcome?

**NO**

**YES**

**YES**

**YES**

**Appendix 5: Coding tool**

| **S.no.** | **Question** | **Options** |
| --- | --- | --- |
| 1. | Exclude | No studies from LMICs  Not relevant due to outcome or intervention |
| 2. | Year | ENTER ‘Year of study’ |
| 3. | Study design | - RCT - RDD - DiD - Propensity score matching - Before and after - Time series - Systematic review |
| 4. | Publication status | - Completed - Ongoing |
| 5. | Population | - Infanthood (<3 years) - Childhood (3-10 years) - Adolescence (10-18 years) - Parents and Caregivers - Teachers/health care staff - Boys/male child - Girls/female child - Pregnant women and girls - Children with disabilities - Chronic physical illness - Children in low income settings - Marginalised group of women - Ethnic minorities - Not children (Women/higher age group) - Rural - Urban - Fathers - Mothers - Child brides |
| 6. | Type of violence | Interpersonal violence   - Intimate partner violence/ violence by immediate family - Community violence |
| 7. | Region | East Asia and Pacific  Europe and Central Asia  North America  Middle East and North Africa  South Asia  Sub-Saharan Africa  Latin America and Caribbean |
| 8. | Conflict affected region | High fragility regions   - Afghanistan - Chad - DRC - Eritrea - Libya - Myanmar - Nigeria - Pakistan - Somalia - South Sudan - Syria - Yemen   Medium fragility regions   - Zimbabwe - Tajikistan - Lebanon - Guinea - Congo, Rep - Haiti - Turkmenistan - Iran - Uzbekistan - Egypt   Low-fragility regions   - Liberia - Angola - Ethiopia - Mali - Bangladesh - Kenya - Madagascar - Nepal - Honduras - Kyrgyz Republic   Neighbours   - Benin - Zambia - Tanzania - Uganda - Rwanda   ENTER ‘Not conflict affected’ |
| 9. | Intervention | Laws, crime and justice   - Law - Crime and justice systems   Norms and values   - Community mobilization programmes - Bystander interventions - Media campaigns including mass media and education   Safe environments   - Making existing environments safe - Creating safe places   Parent, child and caregiver support   - Parent-training and education - Maternal/Paternal mental health - Peer/relationship training   Income and economic strengthening   - Broad-based social protection (Economic transfers) - Income generating or savings/credit interventions - Insurance and welfare schemes   Response and support services   - Counselling and therapeutic approaches - Screening and training - Children in care - Media and communication   Education and life skills   - Gender transformative approaches - Life and social skills training |
| 10. | Outcome | Violence   - Sexual - Physical - Emotional/ psychological (financial abuse)   Norms, values, beliefs and attitudes   - Belief on parenting practices - Gender roles, attitudes and social norms - Delinquent, violent and other risk-taking behaviour - Empowerment   Health   - Substance abuse - Child development and child mental health - Maternal mental health - Morbidity and mortality - Sexual and reproductive health   Safety and risk factors for other harms   - Social isolation - Female Genital mutilation (FGM) and child marriage - Child labour/ trafficking - Safe environment/ spaces   Economic and social   - Poverty and food security - Employment and labour force participation - Savings and credit - Social inclusion and gender equity - Social discrimination   Cost analysis   - Cost-effectiveness - Cost-benefit   Education   - School enrolment and attendance - School performance - WASH and infrastructure - Gender roles and life skills |
| 11. | Funding agency/ Donors | ENTER ‘Name of funding agency’ |
| 12. | Systematic review confidence | AMSTAR 2 tool (Criteria 1-16 [given in Appendix 6] and final scoring)  Low confidence  Medium confidence  High confidence  Ongoing |
| 13. | Critical appraisal for impact evaluation | Critical appraisal tool (Criteria 1-7 [given in Appendix 7] and final scoring)  Low confidence  Medium confidence  High confidence  Ongoing |
| 14. | Ethical tool for impact evaluation | Ethical tool (Criteria 1-9 [given as appendix 8] and final scoring  Adequate ethical standards  Inadequate ethical standards |

**Appendix 6: AMSTAR 2**

| **S.no.** | **Question** | **Option** |
| --- | --- | --- |
| 1. | Did the research questions and inclusion criteria for the review include the components of PICO? | Yes  No |
| 2.  ** | Did the report of the review contain an explicit statement that the review methods were established prior to the conduct of the review and did the report justify any significant deviations from the protocol? | Yes  Partial Yes  No |
| 3. | Did the review authors explain their selection of the study designs for inclusion in the review? | Yes  No |
| 4.  ** | Did the review authors use a comprehensive literature search strategy? | Yes  Partial Yes  No |
| 5. | Did the review authors perform study selection in duplicate? | Yes  No |
| 6. | Did the review authors perform data extraction in duplicate? | Yes  No |
| 7.  ** | Did the review authors provide a list of excluded studies and justify the exclusions? | Yes  Partial Yes  No |
| 8. | Did the review authors describe the included studies in adequate detail? | Yes  Partial Yes  No |
| 9.  ** | Did the review authors use a satisfactory technique for assessing the risk of bias (RoB) in individual studies that were included in the review? | Yes  Partial Yes  No  Includes only NRSI/ RCTs |
| 10. | Did the review authors report on the sources of funding for the studies included in the review? | Yes  No |
| 11.  ** | If meta-analysis was performed did the review authors use appropriate methods for statistical combination of results? | Yes  No  No meta-analysis conducted |
| 12. | If meta-analysis was performed, did the review authors assess the potential impact of RoB in individual studies on the results of the meta-analysis or other evidence synthesis? | Yes  No  No meta-analysis conducted |
| 13.  ** | Did the review authors account for RoB in individual studies when interpreting/ discussing the results of the review? | Yes  No |
| 14. | Did the review authors provide a satisfactory explanation for, and discussion of, any heterogeneity observed in the results of the review? | Yes  No |
| 15.  ** | If they performed quantitative synthesis did the review authors carry out an adequate investigation of publication bias (small study bias) and discuss its likely impact on the results of the review? | Yes  No  No meta-analysis conducted |
| 16. | Did the review authors report any potential sources of conflict of interest, including any funding they received for conducting the review? | Yes  No |

****Critical item**

**Rating overall confidence in the results of the review**

***High***

No or one non-critical weakness: the systematic review provides an accurate and comprehensive summary of the results of the available studies that address the question of interest

***Moderate***

More than one non-critical weakness*: the systematic review has more than one weakness but no critical flaws. It may provide an accurate summary of the results of the available studies that were included in the review

***Low***

One critical flaw with or without non-critical weaknesses: the review has a critical flaw and may not provide an accurate and comprehensive summary of the available studies that address the question of interest

***Critically low***

More than one critical flaw with or without non-critical weaknesses: the review has more than one critical flaw and should not be relied on to provide an accurate and comprehensive summary of the available studies

*Multiple non-critical weaknesses may diminish confidence in the review and it may be appropriate to move the overall appraisal down from moderate to low confidence

**Appendix 7: Critical appraisal tool**

| **Item** | | **Point in time (where applicable)** | **Rating** |
| --- | --- | --- | --- |
| **1a** | **Study design (Potential confounders taken into account)** | End of intervention | High confidence: RCT, RDD, ITT, instrumental variable  Medium confidence: DiD with matching, PSM  Low confidence: other matching |
| **1b** | **Study design (Potential confounders taken into account)** | Longest follow up (if applicable) | Study design may change at post endline follow up, usually loss of RCT as control becomes treated. Same codes as 1a |
| **2** | **Masking or blinding (RCTs only)** |  | High confidence: any blinding or any mention of blinding  Medium confidence: no blinding  Low confidence is not used for this item |
| **3** | **Power calculations are reported** |  | High confidence: any mention of power calculations as basis for sample size  Medium confidence: no mention of power calculations  Low confidence is not used for this item |
| **4a** | **Losses to follow up are presented and acceptable*** | End of intervention | High: attrition within IES bounds  Medium: attrition close to IES bounds  Low: attrition not reported or attrition outside IES bounds  N/A for ex post studies |
| **4b** | **Losses to follow up are presented and acceptable*** | Longest follow up (if applicable) | High: attrition within IES bounds  Medium: attrition close to IES bounds  Low: attrition not reported or attrition outside IES bounds  N/A for ex post studies |
| **5** | **Intervention if clearly defined** |  | High confidence: intervention clearly and fully described  Medium confidence: brief description of intervention  Low confidence: intervention named but not described, or not named |
| **6** | **Outcome measures are clearly defined and reliable** |  | High confidence: outcome measure clearly and fully described, preferably with reference to validation  Medium confidence: brief description of outcome  Low confidence: outcome named but not described |
| **7** | **Baseline balance (N.A. for before versus after)** |  | High confidence: RCT or baseline balance report and satisfactory (imbalance on 5 or less than 5 percent)  Medium confidence: Imbalance between 5-10 percent  Low confidence: Baseline balance not reported, or reported and lack of balance on 10 or more than 10 percent |
|  | **Overall confidence in study findings** | End of intervention | Lowest rating across items 1a, 4a, 6 and 7 |
|  | **Overall confidence in study findings** | Longest follow up (if applicable) | Lowest rating across items 1b, 4b, 6 and 7 (N/A if 1b and 4b N/A) |

* Maximum acceptable rate of differential attrition for each overall attrition rate (Source: Deke, Sama-Miller & Hershey, 2015)

**Appendix 8: Ethics coding tool**

‘Ethics’ can be defined as a system or code of moral values that provides rules and standards of conduct. The three primary ethical principles that should guide all inquiries involving human beings (including methods used to collect information) are as follows:

1) Respect for persons, which relates to respecting the autonomy and self-determination of participants, and protecting those who lack autonomy, including by providing security from harm or abuse.

2) Beneficence, a duty to safeguard the welfare of people/communities involved, which includes minimizing risks and assuring that benefits outweigh risks.

3) Justice, a duty to distribute benefits and burdens fairly.

The nine item tool has 5 critical and 4 non-critical items (rating given below):

Ethical adequacy Tool:

|  | **Question** | **Response** |
| --- | --- | --- |
| 1 | Does the study have an ethical committee approval? * | Yes/ No / Can’t say |
| 2 | Does the study mention about training given to data collectors? * | Yes/ No / Can’t say |
| 3 | Does the study mention about informed consent from participants/ providing information about study to participants? * | Yes/ No / Can’t say |
| 4 | Was the intervention given/ considered to control arm after the study was complete/ as a part of study design? | Yes/ No / Can’t say |
| 5 | Does the study declare researcher interest and funding?* | Yes/ No / Can’t say |
| 6 | Does the study mention how confidentiality/ anonymity of participants was preserved?* | Yes/ No / Can’t say |
| 7 | Does the study mention about consideration given to respondents’ willingness to disclose victimization? | Yes/ No / Can’t say |
| 8 | Does the study mention about dissemination policy (right to result, potential risks, purpose and benefit)? | Yes/ No / Can’t say |
| 9 | Does the study mention about engagement with local community/ government agencies/ research partners in conflict settings? | Yes/ No / Can’t say |
|  |  |  |

*Critical item

Rating ‘Strong ethical standards’ if response is yes for more than or equal to 3 critical items. Rating ‘Moderate ethical standards’ if response is yes for at least 2 critical items.

Rating ‘Low ethical standards’ if critical item is marked as ‘yes’ for one or no critical item(s)

**Appendix 9- List of funding agencies**

| **S.no.** | | **Funding agency/Donors** | | **Frequency** | |
| --- | --- | --- | --- | --- | --- |
| 1 | | Alexander von Humboldt Foundation | | 1 | |
| 2 | | American Jewish World Service | | 1 | |
| 3 | | Anglo Platinum | | 1 | |
| 4 | | Anonymous donor | | 2 | |
| 5 | | Australian AID | | 1 | |
| 6 | | Australian Government | | 1 | |
| 7 | | Australian Research Council | | 1 | |
| 8 | | AVSI Foundation | | 1 | |
| 9 | | Bernard Van Leer foundation | | 3 | |
| 10 | | Bill and Melinda Gates Foundation | | 3 | |
| 11 | | Brazilian Ministry of Health | | 2 | |
| 12 | | Canadian Institutes of Health Research | | 1 | |
| 13 | | Consultative Group for International Agricultural Research (CGIAR) | | 1 | |
| 14 | | Chile Ministry of Health | | 1 | |
| 15 | | Comic Relief (UK) | | 1 | |
| 16 | | David and Lucile Packard Foundation | | 1 | |
| 17 | | Department of Biotechnology, Government of India | | 1 | |
| 18 | | Deutsche Forschungsgemeinschaft & Ein Herz fur Kinder foundation | | 1 | |
| 19 | | Economic and Social Research Council (ESRC) | | 3 | |
| 20 | | Eliminating Child Labour in Tobacco Growing Foundation (ECLT) | | 1 | |
| 21 | | European Union | | 7 | |
| 22 | | Ford Foundation | | 1 | |
| 23 | | Foundation Centrum | | 1 | |
| 24 | | Global Center Grant Acquisition & Management/Innovation | | 1 | |
| 25 | | Global Development Network | | 2 | |
| 26 | | Government of Indonesia | | 1 | |
| 27 | | GTZ Supra Regional Project | | 1 | |
| 28 | | Henry J Kaiser Family Foundation | | 1 | |
| 29 | | Hewlett Foundation | | 3 | |
| 30 | | Ian Potter Foundation | | 1 | |
| 31 | | ICF International | | 1 | |
| 32 | | IKEA Foundation | | 1 | |
| 33 | | Ilifa Labantwana Fund & South African National Lottery Distribution Trust Fund | | 1 | |
| 34 | | Innovations for Poverty Action (IPA) | | 1 | |
| 35 | | International Development Research Centre (Canada) | | 1 | |
| 36 | | International Growth Centre | | 1 | |
| 37 | | International HIV/AIDS alliance | | 1 | |
| 38 | | International Initiative for Impact Evaluation (3IE) | | 4 | |
| 39 | | International Labour Organisation (ILO) | | 3 | |
| 40 | | Jacobs Foundation | | 1 | |
| 41 | | John D. and Catherine T. MacArthur Foundation | | 4 | |
| 42 | | Abdul Latif Jameel Poverty Action Lab (JPAL) | | 1 | |
| 43 | | Know Violence in Childhood | | 1 | |
| 44 | | LOGiCA trust fund | | 2 | |
| 45 | | Manassehâ Children (US based NGO) | | 1 | |
| 46 | | Medical Research Council (MRC) | | 4 | |
| 47 | | National Institute for Health Research (UK) | | 2 | |
| 48 | | National Institutes of Health (NIH) (US) | | 7 | |
| 49 | | No financial support | | 6 | |
| 50 | | Norwegian Knowledge Centre for Violence and Traumatic Stress Studies | | 1 | |
| 51 | | Novo Foundation & Sigrid Rausing Trust | | 1 | |
| 52 | | Oak Foundation | | 7 | |
| 53 | | Petra Perdana Berhad (Malaysian Corporation) | | 1 | |
| 54 | | Philip Leverhulme Trust | | 1 | |
| 55 | | Population Council | | 4 | |
| 54 | | Queensland University | | 1 | |
| 57 | | Robert Wood Johnson foundation | | 1 | |
| 58 | | Rockfeller Foundation | | 1 | |
| 59 | | Royal Embassy of Netherlands | | 1 | |
| 60 | | SÃo Paulo Research Foundation (FAPESP) | | 1 | |
| 61 | | Save the Children | | 2 | |
| 62 | | Scottish child abuse inquiry | | 1 | |
| 63 | | Sexual Violence Research Initiative (SVRI), South Africa | | 1 | |
| 64 | | Spanish Impact Evaluation Fund | | 1 | |
| 65 | | Swedish Board of Health and Welfare | | 1 | |
| 66 | | Swedish International Development Cooperation Agency (Sida) | | 1 | |
| 67 | | The John Fell Fund | | 2 | |
| 68 | | The Nike Foundation | | 2 | |
| 69 | | The Wellcome Trust | | 4 | |
| 70 | | TUBITAK SOBAG | | 1 | |
| 71 | | UBS Optimus Foundation | | 1 | |
|  | |  | |  | |
| 73 | | Ujamaa-Africa (NGO) | | 1 | |
| 74 | | Uludag University Scientific Research Projects | | 1 | |
| 75 | | UNFPA and UN Fund | | 1 | |
| 76 | | UNICEF | | 7 | |
| 77 | | United Kingdom's Department for International Development (DFID) | | 15 | |
| 78 | | United States Agency for International Development (USAID) | | 13 | |
| 79 | | United States Centers for Disease Control and Prevention (CDC) | | 1 | |
| 80 | | United States Department of Labor | | 2 | |
| 81 | | University of Konstanz | | 1 | |
| 82 | | Unsupported/no information | | 39 | |
|  | |  | |  | |
| 84 | | US Department of State Bureau for Population, Migration and Refugees | | 1 | |
| 85 | | WHO | | 3 | |
| 86 | | World Bank | | 12 | |
| 87 | | World Food Program | | 1 | |
| 88 | | World Vision | | 1 | |
| 89 | | Yale University Institution for Social and Policy | | 1 |  |

**Appendix 10: List of included studies**

1. Abdulmalik J, Ani C, Ajuwon AJ, Omigbodun O. Effects of problem-solving interventions on aggressive behaviours among primary school pupils in Ibadan, Nigeria. Child Adolesc Psychiatry Ment Health [Internet]. 2016 Feb;10 (1):31. Available from: https://capmh.biomedcentral.com/articles/10.1186/s13034-016-0116-5
2. Dammert AC, De Hoop J, Mvukiyehe E, Rosati FC. Effects of public policy on child labor: Current knowledge, gaps and implications for program design. World Development. 2018 Oct 1;110:104-23;
3. Adelufosi A, Arikpo D, Aquaisua E, Meremikwu ML. Cognitive behavioural therapy (CBT) for depression or anxiety disorders in women and girls living with female genital mutilation. Pub Med [Internet]. 2017 Feb;56–9. Available from: https://www.ncbi.nlm.nih.gov/pubmed/28164288
4. Alam A, Baez J, X DC. Does cash for school influence young women’s behavior in the longer term? Evidence from Pakistan [Internet]. World Bank; 2011 May. Available from: https://elibrary.worldbank.org/doi/abs/10.1596/1813-9450-5669
5. Alan C, Hollie D, Fiona C. A Systematic Review of Reviews of the Outcome of Noninstitutional Child Maltreatment. SAGE Jounals [Internet]. 2018 Sep; Available from: https://doi.org/10.1177/1524838018801334
6. Alatas V. Program Keluarga Harapan: impact evaluation of Indonesia’s Pilot Household Conditional Cash Transfer Program. World Bank [Internet]. 2011 Jun; Available from: https://documents.worldbank.org/en/publication/documents-reports/documentdetail/589171468266179965/program-keluarga-harapan-impact-evaluation-of-indonesias-pilot-household-conditional-cash-transfer-program
7. Ali A, Mirza MS, Rauf M. The Effectiveness of Training Program in Changing Teachers’ Behavior Regarding Inflicting Corporal Punishment. J Manag Sci Vol VIII Number [Internet]. 2014;1:98. Available from: http://www.qurtuba.edu.pk/jms/default_files/JMS/8_1/JMS_January_June2014_97-102.pdf
8. Altafim ERP, M LMB. Universal violence and child maltreatment prevention programs for parents: A systematic review. Psychosoc Interv [Internet]. 2016 Apr;25(1):27. Available from: https://www.sciencedirect.com/science/article/pii/S1132055915000502
9. Amin S. Empowering adolescent girls in rural Bangladesh: Kishori Abhijan [Internet]. 2011 May. Available from: https://www.popcouncil.org/uploads/pdfs/TABriefs/13_KishoriAbhijan.pdf
10. Amin S, RT N. Impact of SAFE intervention on sexual and reproductive health and rights and violence against women and girls in Dhaka slums. 2014 Dec; Available from: https://www.popcouncil.org/uploads/pdfs/2014PGY_SAFE-Report.pdf
11. Arango DJ, Ellsberg M, Morton M, Gennari F, Kiplesund S. Interventions to prevent or reduce violence against women and girls: a systematic review of reviews. World Bank [Internet]. 2014 Jan; Available from: http://documents.worldbank.org/curated/en/700731468149970518/Interventions-to-prevent-or-reduce-violence-against-women-and-girls-a-systematic-review-of-reviews
12. Ashburn K, Kerner B, Ojamuge D, Lundgren R. Evaluation of the Responsible, Engaged, and Loving (REAL) Fathers Initiative on Physical Child Punishment and Intimate Partner Violence in Northern Uganda. Prev Sci [Internet]. 2016 Oct;18(7):854–864. Available from: http://dx.doi.org/10.1007/s11121-016-0713-9
13. Atienzo EE, Baxter SK, Kaltenthaler E. Interventions to prevent youth violence in Latin America: a systematic review. Int J Public Health [Internet]. 2016 Oct;62(1):15–29. Available from: https://www.ncbi.nlm.nih.gov/pmc/articles/PMC5288433/
14. Austrian K, Muthengi E, Mumah J, Soler-Hampejsek E, Kabiru CW, Abuya B, et al. The adolescent girls initiative-Kenya (AGI-K): study protocol. BMC Public Health [Internet]. 2016 Dec;16(1). Available from: https://bmcpublichealth.biomedcentral.com/articles/
15. Babalola S, Brasington A, Agbasimalo A, Helland A, Nwanguma E, Onah N. Impact of a communication programme on female genital cutting in eastern Nigeria. Wiley Online Libr [Internet]. 2006 Sep;11(10):1594–603. Available from: https://doi.org/10.1111/j.1365-3156.2006.01701.x
16. Bacchus LJ, Colombini M, M CU, Howarth E, Gardner F, Annan J, et al. Exploring opportunities for coordinated responses to intimate partner violence and child maltreatment in low and middle income countries: a scoping review. Psychol Heal Med [Internet]. 2017 Mar;22(sup1):135–65. Available from: https://www.ncbi.nlm.nih.gov/pubmed/28150500
17. Baiocchi M, Omondi B, Langat N, Boothroyd DB, Sinclair J, Pavia L, et al. A behavior-based intervention that prevents sexual assault: The results of a matched-pairs, cluster-randomized study in Nairobi, Kenya. Prev Sci [Internet]. 2017 Oct;18(7):818–27. Available from: https://www.ncbi.nlm.nih.gov/pubmed/27562036
18. Baird SJ, Chirwa E, J DH, Özler B. Girl power: cash transfers and adolescent welfare. Evidence from a cluster-randomized experiment in Malawi [Internet]. National Bureau of Economic Research; 2013 Sep. Available from: https://www.nber.org/papers/w19479
19. Baird S, Chirwa E, McIntosh C. The Short-Term Impacts of a Schooling Conditional Cash Transfer Program on the Sexual Behavior of Young Women. 2010; Available from: https://www.ncbi.nlm.nih.gov/pubmed/19946887

20. Baker-Henningham H, Vera-Hernández M, Alderman H, Walker S. Irie Classroom Toolbox: a study protocol for a cluster-randomised trial of a universal violence prevention programme in Jamaican preschools. BMJ Open [Internet]. 2016 Jan;6(5):e012166. Available from: https://bmjopen.bmj.com/content/6/5/e012166

21. Bandiera O, Buehren N, Burgess R, Goldsteinl M, Gulesci M, Rasul I, et al. Empowering Adolescent Girls: Evidence from a Randomized Control Trial in Uganda. eLibrary [Internet]. 2012 Dec; Available from: https://elibrary.worldbank.org/doi/abs/10.1596/25529

22. Barker G. Promoting gender-equity among young Brazilian men as an HIV prevention strategy. 2006; Available from: https://hivhealthclearinghouse.unesco.org/library/documents/promoting-gender-equity-among-young-brazilian-men-hiv-prevention-strategy

23. Barlow J, MacMillan H, Macdonald G, Bennett C, SK L. Psychological interventions to prevent recurrence of emotional abuse of children by their parents. Cochrane Database Syst Rev [Internet]. 2013;(9). Available from: http://dx.doi.org/10.1002/14651858.CD010725

24. Bastagli F, Hagen-Zanker J, V HLB, Sturge G, Schmidt T, Pellerano L. Cash transfers: what does the evidence say? A rigorous review of programme impact and of the role of design and implementation features. 2016 Jul;300. Available from: https://www.odi.org/publications/10505-cash-transfers-what-does-evidence-say-rigorous-review-impacts-and-role-design-and-implementation

25. Beattie TS, Bhattacharjee P, Isac S, Davey C, Javalkar P, Nair S, et al. Supporting adolescent girls to stay in school, reduce child marriage and reduce entry into sex work as HIV risk prevention in north Karnataka, India: protocol for a cluster randomised controlled trial . BMC Public Health [Internet]. 2015 Mar; Available from: https://bmcpublichealth.biomedcentral.com/articles/10.1186/s12889-015-1623-7

26. Ben P, William T, Hospital P, Chelmsford, UK. Psychoanalytic/Psychodynamic Psychotherapy for Sexually Abused Children and Adolescents: A Systematic Review. Res Soc Work Pract [Internet]. 2014;24(4):389–99. Available from: http://www.epistemonikos.org/documents/430a3dd56cb49775690048ddabede8d1be2d3deb

27. Bourey C, Williams W, Bernstein EE, Stephenson d R. Systematic review of structural interventions for intimate partner violence in low-and middle-income countries: organizing evidence for prevention. BMC Public Health [Internet]. 2015 Nov;15:1165. Available from: https://www.ncbi.nlm.nih.gov/pmc/articles/PMC4657265/

28. Breakthrough. Baseline Report on Impact Evaluation of Breakthrough’s Early Marriage Campaign [Internet]. 2013. Available from: https://inbreakthrough.org/wp-content/uploads/2018/11/Baseline-Report-Early-Marriage.pdf

29. Buchmann N, Field E, Glennerster R, Nazneen S, Pimkina S, Sen I. Power vs money: Alternative approaches to reducing child marriage in Bangladesh, a randomized control trial. J-Pal [Internet]. 2017; Available from: https://www.povertyactionlab.org/sites/default/files

30. Buller AM, Peterman A, Ranganathan M, Bleile A, Hidrobo M, Heise L. A mixed-method review of cash transfers and intimate partner violence in low and middle-income countries. Off Res [Internet]. 2018 Sep;33(2):218–258. Available from: https://academic.oup.com/wbro/article/33/2/218/5091868

31. Candace M, Maxton T. Cash Transfers and Children’s Education and Labour among Malawi’s Poor. Dev Policy Rev [Internet]. 2012;30(4):499–522. Available from: https://onlinelibrary.wiley.com/doi/abs/10.1111/j.1467-7679.2012.00586.x

32. Cardoso E, AP S. The Impact of Cash Transfers on Child Labor and School Attendance in Brazil. IDEAS [Internet]. 2004; Available from: https://ideas.repec.org/p/van/wpaper/0407.html

33. Cecen-Erogul AR, O KH. The Effectiveness of Psycho-educational School-based Child Sexual Abuse Prevention Training Program on Turkish Elementary Students. Educ Sci Theory Pract [Internet]. 2013;13(2):725–9. Available from: https://files.eric.ed.gov/fulltext/EJ1017300.pdf

34. Celedón L, Luis, Bustos S, Helia, Castro R, Katherinne. Maltreatment in early childhood: a scoping review of prevention, detection and treatment. Res Gate [Internet]. 2013 Oct;184:9–10. Available from: https://www.researchgate.net/publication/265341821_Prevention_of_violence_abuse_and_neglect_in_early_childhood_A_review_of_the_literature_on_research_policy_and_practice

35. Chamroonsawasdi K. Gender roles, physical and sexual violence prevention in primary extend to secondary school in Samutsakorn Province, Thailand. PubMed [Internet]. 2010 Mar;93(3):358–65. Available from: https://www.ncbi.nlm.nih.gov/pubmed/20420112

36. Chaudhury S, Brown FL, CM K, Mukunzi S, Nyir, agijimana B, et al. Exploring the potential of a family-based prevention intervention to reduce alcohol use and violence within HIV-affected families in Rwanda. Pub Med [Internet]. 2016 Mar;118–29. Available from: https://www.ncbi.nlm.nih.gov/pubmed/27392007

37. Chege J, Askew I, Igras S, Muteshi-Strachan J. Testing the effectiveness of integrating communitybased approaches for encouraging abandonment of female genital cutting into CARE’ reproductive health programs in Ethiopia and Kenya. ResearchGate [Internet]. 2004 Jan; Available from: https://www.researchgate.net/publication/239536974_Testing_the_Effectiveness_of_Integrating_Community-Based_Approaches_for_Encouraging_Abandonment_of_Female_Genital_Cutting_into_CARE’s_Reproductive_Health_Programs_in_Ethiopia_and_Kenya

38. Chen M, Chan KL. Effects of parenting programs on child maltreatment prevention: A meta-analysis. Trauma, Violence, Abus [Internet]. 2015 Jan;17(1):88–104. Available from: https://www.ncbi.nlm.nih.gov/pubmed/25573846

39. Christopher B, Green E, Annan J, Jamison J. "Building Women’s Economic and Social Empowerment Through Enterprise An Experimental Assessment of the Women’s Income Generating Support (WINGS) Program in Uganda. World Bank [Internet]. 2013 Apr;1(1). Available from: https://documents.worldbank.org/en/publication/documents-reports/documentdetail/927131468316473189/building-womens-economic-and-social-empowerment-through-enterprise-an-experimental-assessment-of-the-womens-income-generating-support-wings-program-in-uganda

40. Christopher M, Alexander B. Child maltreatment prevention: a systematic review of reviews. Bull World Health Organ [Internet]. 2009 Feb;87:353–61. Available from: https://www.scielosp.org/pdf/bwho/2009.v87n5/353-361

41. Clea S, Benjamin O, Jake S, Carolinah G, Lee P, Munyae M, et al. Rape prevention through empowerment of adolescent girls. PubMed [Internet]. 2014 May;133(5):peds-2013. Available from: https://www.ncbi.nlm.nih.gov/pubmed/24733880

42. Cluver LD, Lachman JM, Ward CL, Gardner F, Peterson T, Hutchings JM, et al. Development of a Parenting Support Program to Prevent Abuse of Adolescents in South Africa: Findings From a Pilot Pre-Post Study. Res Soc Work Pract [Internet]. 2016 Feb;27(7):758–66. Available from: https://journals.sagepub.com/doi/abs/10.1177/1049731516628647

43. Cluver L, Meinck F, Shenderovich Y, CL W, RH R, Redfern A, et al. A parenting programme to prevent abuse of adolescents in South Africa: study protocol for a randomised controlled trial. NCBI [Internet]. 2016;17(1):328. Available from: https://www.ncbi.nlm.nih.gov/pmc/articles/PMC4950110/

44. Cluver L, Meinck F, Yakubovich A, Doubt J, Redfern A, Ward C, et al. Reducing child abuse amongst adolescents in low- and middle-income countries: A pre-post trial in South Africa. PubMed [Internet]. 2016 Jul;79(2):179–93. Available from: https://www.ncbi.nlm.nih.gov/pmc/articles/PMC5137206/

45. Colucci E, Hassan G. Prevention of domestic violence against women and children in low-and-middle-income-countries. Pub Med [Internet]. 2014 Sep;27(5):350–7. Available from: https://www.ncbi.nlm.nih.gov/pubmed/25033276

46. Cooper PJ, Tomlinson M, Swartz L, L, man M, Molteno C, et al. Improving quality of mother-infant relationship and infant attachment in socioeconomically deprived community in South Africa: randomised controlled trial. Bmj [Internet]. 2009 May;338:b974. Available from: https://www.bmj.com/content/338/bmj.b1858

47. Coore DC, Reece JA, Shakespeare-Pellington S. The prevention of violence in childhood through parenting programmes: a global review. Psychol Heal Med [Internet]. 2017 Jan;22(1). Available from: https://doi.org/10.1080/13548506.2016.1271952

48. Courtin E, Layte R, Avendano M, Allchin E. Interventions to reduce or prevent exposure to adverse experiences in childhood (ACEs): a systematic review. 2019;

49. Davis B, Daidone S. The Impact of Ghana’s LEAP Programme. IDEAS [Internet]. 2014;271. Available from: https://ideas.repec.org/p/ipc/opager/271.html

50. dDe Hoop J, Rosati FC. Cash transfers and child labor. The World Bank Research Observer. 2014 Aug 1;29(2):202-3451.

51. Denison E, Berg RC, Lewin S, Fretheim A. Effectiveness of interventions designed to reduce the prevalence of female genital mutilation/cutting. NCBI [Internet]. 2011; Available from: https://www.ncbi.nlm.nih.gov/books/NBK464894/

52. Derakhshanpour F, Hajebi A, Panaghi L, Ahmadabadi Z. Effectiveness of psychosocial interventions in abused children and their families. Med J Islam Repub Iran [Internet]. 2017;31:49. Available from: https://www.ncbi.nlm.nih.gov/pmc/articles/PMC5804423/ https://www.ncbi.nlm.nih.gov/pmc/articles/PMC5804423/pdf/mjiri-31-49.pdf https://www.ncbi.nlm.nih.gov/pmc/articles/PMC5804423/

53. Devries KM, Knight L, Child JC, Mirembe A, Nakuti J, Jones R, et al. The Good School Toolkit for reducing physical violence from school staff to primary school students: a cluster-randomised controlled trial in Uganda. Lancet Glob Heal [Internet]. 2015 Jul;3(7):e378–86. Available from: https://www.ncbi.nlm.nih.gov/pubmed/26087985

54. Dhar D, Jain T, Jayach, ran S. Reshaping Adolescents’ Gender Attitudes: Evidence from a School-Based Experiment in India [Internet]. 2018. Available from: https://www.povertyactionlab.org/fr/node/9759

55. Dickson K, Bangpan M. Providing access to economic assets for girls and young women in low-and-lower middle-income countries: A systematic review. 2012; Available from: https://asiawomen.org.sg/docs/Providing access to eco

56. Diop NJ, Faye MM, Moreau A, Cabral J, Benga H, Cissé F, et al. The TOSTAN program: evaluation of a community based education program in Senegal. USAID [Internet]. 2004 Aug; Available from: https://www.k4health.org/sites/default/files/TOSTAN program_Evaluation of CommBased Edu Pgm_Senegal.pdf

57. Diop NJ, Traoré F, Diallo H, Traoré O, Touré AH, Diallo Y, et al. Study of the effectiveness of training Malian social and health agents in female genital cutting issues and in educating their clients. 2000 Dec; Available from: http://citeseerx.ist.psu.edu/viewdoc/download?doi=10.1.1.398.4578&rep=rep1&type=pdf

58. Doğan A, Keser E, Şen Z, Yanagida T, Gradinger P, Strohmeier D. Evidence based bullying prevention in turkey: Implementation of the ViSC social competence Program. Int J Dev Sci [Internet]. 2017 Dec;11(3–4):93–108. Available from: https://www.researchgate.net/publication/322142193_Evidence_Based_Bullying_Prevention_in_Turkey_Implementation_of_the_ViSC_Social_Competence_Program

59. Dubois S, Job-Spira N, Lamour M, Ferron C, Leboube F, Lebovici S. Implementation and evaluation of a program of prevention against abuse in at risk infants. Arch Fr Pediatr [Internet]. 2000 Jan;48(3):189‐200. Available from: https://www.cochranelibrary.com/central/doi/10.1002/central/CN-00075996/full

60. Dunbar MS, Dufour K, Lambdin B, Mudekunye-Mahaka I Nhamo D. The SHAZ! Project: Results from a Pilot Randomized Trial of a Structural Intervention to Prevent HIV among Adolescent Women in Zimbabwe. PLoS One [Internet]. 2014 Nov; Available from: https://journals.plos.org/plosone/article?id=10.1371/journal.pone.0113621

61. Ellsberg M, Arango DJ, Morton M, Gennari F, Kiplesund S, Contreras M, et al. Prevention of violence against women and girls: what does the evidence say? 2015 Apr;385(9977):1555–66. Available from: https://doi.org/10.1016/S0140-6736(14)61703-7

62. Emma F, Kerr-Wilson A, Lang J. What works to prevent violence against women and girls? Evidence Review of interventions to prevent violence against women and girls . GOVUK [Internet]. 2014 Jan; Available from: https://www.gov.uk/dfid-research-outputs/what-works-to-prevent-violence-against-women-and-girls-evidence-review-of-interventions-to-prevent-violence-against-women-and-girls

63. Ertl V, Pfeiffer A, Schauer E, Elbert T, Neuner F. Community-Implemented Trauma Therapy for Former Child Soldiers in Northern Uganda: A Randomized Controlled Trial. 2011; Available from: https://jamanetwork.com/journals/jama/article-abstract/1104179

64. Erulkar AS, Muthengi E. Evaluation of Berhane Hewan: a program to delay child marriage in rural Ethiopia. Int Perspect Sex Reprod Health [Internet]. 2009 Mar;6–14. Available from: https://www.guttmacher.org/journals/ipsrh/2009/03/evaluation-berhane-hewan-program-delay-child-marriage-rural-ethiopia

65. Euser S, Alink LR, Stoltenborgh M, Bakermans-Kranenburg MJ, H van IjM. A gloomy picture: a meta-analysis of randomized controlled trials reveals disappointing effectiveness of programs aiming at preventing child maltreatment. 2015;

66. Edmonds EV, Shrestha M. You get what you pay for: Schooling incentives and child labor. Journal of Development Economics. 2014 Nov 1;111:196-21167. Falb KL, Annan J, Kpebo D, Cole H, Willie T, Xuan Z, et al. Differential Impacts of an Intimate Partner Violence Prevention Program Based on Child Marriage Status in Rural Côte d’Ivoire. 2015 Nov; Available from: https://www.jahonline.org/article/S1054-139X(15)00301-8/fulltext

68. Rossi M. Rossi M, Rosati FC. Impact of school quality on child labor and school attendance: the case of CONAFE Compensatory Education Program in Mexico. Understanding Chldren's Work Programme Working Paper, February. 2007 Feb.

69. Citak Tunc G, Gorak G, Ozyazicioglu N, Ak B, Isil O, Vural P. Preventing child sexual abuse: Body safety training for young children in Turkey. Journal of child sexual abuse. 2018 May 19;27(4):347-64.

70. Gardner F, Montgomery P, Knerr W. Transporting evidence-based parenting programs for child problem behavior (age 3–10) between countries: Systematic review and meta-analysis. J Clin Child Adolesc Psychol [Internet]. 2016 Mar;45(6):749–62. Available from: https://www.tandfonline.com/doi/full/10.1080/15374416.2015.1015134

71. Green EP, Blattman C, Jamison J, Annan J. Women’s entrepreneurship and intimate partner violence: A cluster randomized trial of microenterprise assistance and partner participation in post-conflict Uganda. PubMed [Internet]. 2015 Jan;177–88. Available from: https://www.ncbi.nlm.nih.gov/pubmed/25875324

72. Habigzang LF, Stroeher FH, Hatzenberger R, Cunha RC, Mda R, Koller SH. Cognitive behavioral group therapy for sexually abused girls. Rev Saude Publica [Internet]. 2009 Aug;43 Suppl 1:70‐78. Available from: https://www.ncbi.nlm.nih.gov/pubmed/19669067

73. Hale DR, Fitzgerald-Yau N, Viner RM. A systematic review of effective interventions for reducing multiple health risk behaviors in adolescence. Pub Med [Internet]. 2014 Mar;104(5):e19–41. Available from: https://www.ncbi.nlm.nih.gov/pubmed/24625172

74. Hallman K, Kelvin E, Ozler B, Seban J, Kuhlik E, Alton C. Girl Empower Intervention: Baseline survey [Internet]. 2016 May. Available from: https://www.rescue.org/sites/default/files/document/825/girlempowerbaselinesurveyreportliberia-23may2016-final1.pdf?platform=hootsuite

75. Hannah T, Jeffrey F, Nancy G, Shannon D. Effectiveness of Interventions, Programs and Strategies for Gender-based Violence Prevention in Refugee Populations: An Integrative Review. PLOS Curr Disasters. 2016;

76. Heise LL. What Works to Prevent Partner Violence? An Evidence Overview. 2011 Dec; Available from: https://www.oecd.org/derec/49872444.pdf

77. Hidrobo M, Peterman A, Heise L. The effect of cash, vouchers, and food transfers on intimate partner violence: evidence from a randomized experiment in Northern Ecuador. Am Econ J Appl Econ [Internet]. 2016;8(3):284–303. Available from: https://www.wfp.org/sites/default/files/IPV-Hidrobo-Peterman_Heise_IPV Ecuador 3 28 14.pdf

78. Hossain M, Zimmerman C, Kiss L, Abramsky T, Kone D, Bakayoko-Topolska M, et al. Working with men to prevent intimate partner violence in a conflict-affected setting: a pilot cluster randomized controlled trial in rural Côte d’Ivoire. BMC Public Health [Internet]. 2014 Dec;14(339). Available from: https://bmcpublichealth.biomedcentral.com/articles/10.1186/1471-2458-14-339

79. Hughes K, Bellis MA, Hardcastle KA, Butchart A, Dahlberg LL, Mercy JA, et al. Global development and diffusion of outcome evaluation research for interpersonal and self-directed violence prevention from 2007 to 2013: A systematic review. Aggress Violent Behav [Internet]. 2014 Dec;19(6):655–62. Available from: https://www.sciencedirect.com/science/article/pii/S1359178914001025

80. De Silva I, Sumarto S. How do educational transfers affect child labour supply and expenditures? Evidence from indonesia of impact and flypaper effects. Oxford Development Studies. 2015 Oct 2;43(4):483-507.

81. Im-Arom C, Rerkswattavorn C, Plitponkarnpim A. Effectiveness of anti-coporal punishment education program among parents in child centres in Bangkok. Inj Prev [Internet]. 2016;22(Suppl 2):A158–9. Available from: https://injuryprevention.bmj.com/content/22/Suppl_2/A158.3 https://injuryprevention.bmj.com/content/injuryprev/22/Suppl_2/A158.3.full.pdf https://injuryprevention.bmj.com/content/22/Suppl_2/A158.3

82. Ismayilova L, Karimli L, Gaveras E, Tô-Camier A, Sanson J, Chaffin J, et al. An integrated approach to increasing women’s empowerment status and reducing domestic violence: Results of a cluster-randomized controlled trial in a West African country. Psychol violence, [Internet]. 2018;8(4):448. Available from: https://psycnet.apa.org/record/2017-33909-001

83. Iyumade OT. Intervention Models of Non-formal Education for the Reintegration of Abused Children in South-Western, Nigeria. 2009 Jul;

84. De Hoop J, Rosati FC. Does promoting school attendance reduce child labor? Evidence from Burkina Faso's BRIGHT project. Economics of Education Review. 2014 Apr 1;39:78-96.

85. Behrman JA, Peterman A, Palermo T. Does keeping adolescent girls in school protect against sexual violence? Quasi-experimental evidence from east and southern Africa. Journal of Adolescent Health. 2017 Feb 1;60(2):184-90.

86. Wagman JA, Gray RH, Campbell JC, Thoma M, Ndyanabo A, Ssekasanvu J, Nalugoda F, Kagaayi J, Nakigozi G, Serwadda D, Brahmbhatt H. Effectiveness of an integrated intimate partner violence and HIV prevention intervention in Rakai, Uganda: analysis of an intervention in an existing cluster randomised cohort. The Lancet Global Health. 2015 Jan 1;3(1):e23-33.

87. Jan S, Ferrari G, CH W, JR H, JC K, Phetla G, et al. Economic evaluation of a combined microfinance and gender training intervention for the prevention of intimate partner violence in rural South Africa. PUB Med [Internet]. 2011 Sep; Available from: https://pubmed.ncbi.nlm.nih.gov/20974751/

88. Kane JC, Murray LK, Cohen J, Dorsey S, Skavenski van Wyk S, Galloway Henderson J, Imasiku M, Mayeya J, Bolton P. Moderators of treatment response to trauma‐focused cognitive behavioral therapy among youth in Zambia. Journal of Child Psychology and Psychiatry. 2016 Oct;57(10):1194-202.

89. Jewkes R, Gibbs A, Jama-Shai N, Willan S, Misselhorn A, Mushinga M, et al. Stepping Stones and Creating Futures intervention: shortened interrupted time series evaluation of a behavioural and structural health promotion and violence prevention intervention for young people in informal settlements in Durban, South Africa. BMC Public Health [Internet]. 2014; Available from: https://bmcpublichealth.biomedcentral.com/track/pdf/10.1186/1471-2458-14-1325

90. Jewkes R, Nduna M, Levin J, Jama N, Dunkle K, Puren A, et al. Impact of stepping stones on incidence of HIV and HSV-2 and sexual behavior in rural South Africa: cluster randomised trial. BMJ [Internet]. 2008 Aug; Available from: https://www.bmj.com/content/337/bmj.a506

91. Lachman JM, Cluver L, Ward CL, Hutchings J, Mlotshwa S, Wessels I, Gardner F. Randomized controlled trial of a parenting program to reduce the risk of child maltreatment in South Africa. Child abuse & neglect. 2017 Oct 1;72:338-51.

92. Joanne S, B ZA, Chinelo A, Geetha R, Powell DG, Léa S. What is the Evidence of the Impact of Initiatives to Reduce Risk and Incidence of Sexual Violence in Conflict and Post-conflict Zones and Other Humanitarian Crises in Lower-and Middle-income Countries?: A Systematic Review [Internet]. EPPI-Centre; 2013. Available from: https://eppi.ioe.ac.uk/cms/Portals/0/PDF reviews and summaries/Conflict zones 2013Spangaro report.pdf?ver=2013-06-19-145907-437

93. Julie P, Lindsay H, Manisha M, Aklilu K, Fabio V, Samuel T. Changing gender norms and reducing intimate partner violence: results from a quasi-experimental intervention study with young men in Ethiopia. Am J Public Health [Internet]. 2015 Jan;105(1):132–7. Available from: https://www.ncbi.nlm.nih.gov/pubmed/25393199

94. Julie P, Wang H, Jennifer A, SL M. Changing gender norms and reducing HIV and violence risk among workers and students in China. J Health Commun [Internet]. 2015 Aug;20(8):869–78. Available from: https://www.ncbi.nlm.nih.gov/pubmed/25950187

95. Gee KA. Reducing child labour through conditional cash transfers: Evidence from nicaragua's red de protección social. Development Policy Review. 2010 Nov;28(6):711-32.

96. Kabeer N, Piza C, Taylor L. Economic impacts of conditional cash transfer programmes: a systematic review and meta-analysis: Journal of Development Effectiveness: Vol 7, No 3 [Internet]. 2015. Available from: https://www.tandfonline.com/doi/abs/10.1080/19439342.2015.1068833

97. Kalamar A, Lee-Rife S, Hindin MJ. Interventions To Prevent Child Marriage Among Young People In Low- And Middle-Income Countries: A Systematic Review Of The Published And Gray Literature. J Adolesc Heal [Internet]. 2016 Sep;59(3):S16–21. Available from: https://www.jahonline.org/article/S1054-139X(16)30161-6/pdf

98. Kaljee L, Zhang L, Langhaug L, Munjile K, Tembo S, Menon A, et al. A randomized-control trial for the teachers’ diploma programme on psychosocial care, support and protection in Zambian government primary schools. Psychol Health Med [Internet]. 2016 Mar;22(4):381–92. Available from: https://www.tandfonline.com/doi/full/10.1080/13548506.2016.1153682?needAccess=true

99. Kanesathasan A, LJ C, Pearson E, SD G, Muherjee S, Malhotra A. Catalyzing Change improving Youth sexual and Reproductive Health Through disha, an Integrated Program in india. ICRW [Internet]. 2008; Available from: https://www.icrw.org/wp-content/uploads/2016/10/Catalyzing-Change-Improving-Youth-Sexual-and-Reproductive-Health-Through-disha-an-Integrated-Program-in-India-DISHA-Report.pdf

100. Kazianga H, Dde W, Alderman H. Educational and Child Labour Impacts of Two Food-for-Education Schemes: Evidence from a Randomised Trial in Rural Burkina Faso. Oxford Acad [Internet]. 2012 Apr; Available from: https://academic.oup.com/jae/article-abstract/21/5/723/784400

101. Keller J, Mboya BO, Sinclair J, Githua OW, Mulinge M, Bergholz L, et al. A 6-week school curriculum improves boys’ attitudes and behaviors related to gender-based violence in Kenya. J Interpers Violence [Internet]. 2017 Feb;32(4):535–57. Available from: https://www.ncbi.nlm.nih.gov/pubmed/26063788

102. Kim J, Mokwena L, Ntlemo E, Dwane N, Noholoza A, Abramsky T, et al. Developing an integrated model for post-rape care and HIV post-exposure prophylaxis in rural South Africa. USAID [Internet]. 2007 Nov; Available from: http://www.svri.org/sites/default/files/attachments/2016-01-18/PNADK615.pdf

103. Yount KM, Krause KH, Miedema SS. Preventing gender-based violence victimization in adolescent girls in lower-income countries: Systematic review of reviews. Social Science & Medicine. 2017 Nov 1;192:1-3.

104. Kilburn KN, Pettifor A, Edwards JK, Selin A, Twine R, MacPhail C, Wagner R, Hughes JP, Wang J, Kahn K. Conditional cash transfers and the reduction in partner violence for young women: an investigation of causal pathways using evidence from a randomized experiment in South Africa (HPTN 068). Journal of the international AIDS society. 2018 Feb;21:e25043.

105. Knerr W, Gardner F, Cluver L. Improving positive parenting skills and reducing harsh and abusive parenting in low-and middle-income countries: A systematic review. Prev Sci [Internet]. 2013 Jan;352–363. Available from: https://link.springer.com/article/10.1007/s11121-012-0314-1

106. Lee‐Rife S, Malhotra A, MG G. What Works to Prevent Child Marriage: A Review of the Evidence. 2012; Available from: https://onlinelibrary.wiley.com/doi/abs/10.1111/j.1728-4465.2012.00327.x

107. Lely JCG, Smid GE, Jongedijk RA, Knipscheer JW, Kleber RJ. The effectiveness of narrative exposure therapy: a review, meta-analysis and meta-regression analysis. Eur J Psychotraumatol. 2019;10.

108. Lester S, Lawrence C, Ward CL. What do we know about preventing school violence? A systematic review of systematic reviews. Psychol Health Med [Internet]. 2017 Jan;22(1):187–223. Available from: https://www.tandfonline.com/doi/full/10.1080/13548506.2017.1282616

109. Litwin A, Perova E, Reynolds S. A conditional cash transfer and Women’s empowerment: Does Bolsa Familia Influence intimate partner violence? Soc Sci Med [Internet]. 2019 Aug; Available from: https://www.researchgate.net/publication/334857795_A_conditional_cash_transfer_and_Women’s_empowerment_Does_Bolsa_Familia_Influence_intimate_partner_violence

110. Murray LK, Skavenski S, Kane JC, Mayeya J, Dorsey S, Cohen JA, Michalopoulos LT, Imasiku M, Bolton PA. Effectiveness of trauma-focused cognitive behavioral therapy among trauma-affected children in Lusaka, Zambia: a randomized clinical trial. JAMA pediatrics. 2015 Aug 1;169(8):761-9.

111. Mathews B, Walsh K, Coe S, Kenny M, Vagenas D. Child protection training for professionals to improve reporting of child abuse and neglect [Cochrane Protocol]. 2015 Jun; Available from: https://www.cochrane.org/CD011775/BEHAV_child-protection-training-for-professionals-to-improve-reporting-of-child-abuse-and-neglect

112. Mehra D, Sarkar A, Sreenath P, Behera J, Mehra S. Effectiveness of a community based intervention to delay early marriage, early pregnancy and improve school retention among adolescents in India. BMC Public Health [Internet]. 2018;18. Available from: https://www.ncbi.nlm.nih.gov/pmc/articles/PMC6000967/ https://www.ncbi.nlm.nih.gov/pmc/articles/PMC6000967/pdf/12889_2018_Article_5586.pdf https://www.ncbi.nlm.nih.gov/pmc/articles/PMC6000967/

113. Meinck F, Little M, Pantelic M, Nittas V, Stöckl H, Orza L. Interventions to prevent and reduce gender-based violence (GBV) among young people living with, or most affected by, HIV in low- and middle-income countries: a systematic review and narrative synthesis. 2018;

114. Melissa M, Claire P, Elizabeth S. Interventions that foster healing among sexually exploited children and adolescents: a systematic review. J Child Sex Abus [Internet]. 2018 Mar;27(4):403–23. Available from: https://doi.org/10.1080/10538712.2018.1477220

115. Menon B, Stoklosa H, K VD, Awerbuch A, Caddell L, Roberts K, et al. Informing Human Trafficking Clinical Care Through Two Systematic Reviews on Sexual Assault and Intimate Partner Violence. NCBI [Internet]. 2018 Nov;1524838018809729. Available from: https://www.ncbi.nlm.nih.gov/pubmed/30453846

116. Merrill KG, Knight L, Namy S, Allen E, Naker D, Devries KM. Effects of a violence prevention intervention in schools and surrounding communities: Secondary analysis of a cluster randomised-controlled trial in Uganda. Child Abuse Negl [Internet]. 2018 Oct;84:182–95. Available from: https://www.ncbi.nlm.nih.gov/pubmed/30114680

117. Miller E, Das M, MCD TDMHV, Nettiksimmons J, O’Connor B, Ghosh S, et al. Evaluation of a gender-based violence prevention program for student athletes in Mumbai, India. PubMed [Internet]. 2014 Mar;29(4):758–78. Available from: https://www.ncbi.nlm.nih.gov/pubmed/24142444

118. Molloy C, Beatson R, Goldfeld S, Harrop C, Perini N. Sustained nurse home visiting programs for disadvantaged families with young children: protocol for a restricted systematic review of program effectiveness and components associated with enhanced health, well-being, and life-course outcomes. Murdoch Child [Internet]. 2012 May; Available from: https://www.rch.org.au/uploadedFiles/Main/Content/ccch/resources_and_publications/Home_visiting_lit_review_RAH_processes_final.pdf

119. Ntaganira J, Brown L, Mock NB. Results of a Community Mentoring Programme for Youth Heads of Household in Rwanda: Effects on Youth Sexual Risk Behaviours and Maltreatment. Rwanda J Heal Sci [Internet]. 2013; Available from: https://www.ajol.info/index.php/rjhs/article/view/85425

120. O’Callaghan P, McMullen J, Shannon C, Rafferty H, Black A. A randomized controlled trial of trauma-focused cognitive behavioral therapy for sexually exploited, war affected Congolese girls. 2013;

121. Oringanje C, Okoro A, Nwankwo O, Meremikwu M. Providing information about female genital mutilation (FGM) to health care providers caring for women and girls living with FGM to improve provider attitude and client satisfaction. PubMed [Internet]. 2017 Feb;136(1):65–71. Available from: https://www.ncbi.nlm.nih.gov/pubmed/28164292

122. Ouoba D, Congo Z Diop NJ, Melching M, Banza B, Guiella G, Baumbarten I. Experience from a Community Based Education Program in Burkina Faso: The Tostan Program. Popul Counc [Internet]. 2006; Available from: https://knowledgecommons.popcouncil.org/departments_sbsr-rh/30/

123. Oveisi S, HE A, MR D, RP M. Primary prevention of parent-child conflict and abuse in Iranian mothers: A randomized-controlled trial. Eur PMC [Internet]. 2010 Mar;34(3):206–13. Available from: http://europepmc.org/abstract/med/20207004

124. De Koker P, Mathews C, Zuch M, Bastien S, Mason-Jones AJ. A systematic review of interventions for preventing adolescent intimate partner violence. Journal of Adolescent Health. 2014 Jan 1;54(1):3-13.

125. Peter V, Paul S. Psychological treatments for orphans and vulnerable children affected by traumatic events and chronic adversity in Sub-Saharan Africa. Jama [Internet]. 2015 Aug;314(5):511–2. Available from: https://jamanetwork.com/journals/jama/article-abstract/2422529

126. Pronyk PM, Hargreaves JR, Kim JC, Morison LA, Phetla G, Watts C, Busza J, Porter JD. Effect of a structural intervention for the prevention of intimate-partner violence and HIV in rural South Africa: a cluster randomised trial. The lancet. 2006 Dec 2;368(9551):1973-83.

127. Prakash R, Beattie T, Bhattacharjee P. Samata intervention to increase secondary school completion and reduce child marriage among adolescent girls from marginalised communities in northern Karnataka. Res Gate [Internet]. 2018 Jul;5. Available from: https://www.researchgate.net/publication/327281380_Samata_intervention_to_increase_secondary_school_completion_and_reduce_child_marriage_among_adolescent_girls_from_marginalised_communities_in_northern_Karnataka

128. Pulerwitz J, Martin S, Mehta M, Castillo T, Kidanu A, Verani F, et al. Promoting Gender Equity for HIV and Violence Prevention: Results from the Male Norms Initiative Evaluation in Ethiopia [Internet]. Path. 2010 Jul. Available from: https://www.path.org/resources/promoting-gender-equity-for-hiv-and-violence-prevention-results-from-the-pepfar-male-norms-initiative-evaluation-in-ethiopia/

129. Berg RC, Denison E. Interventions to reduce the prevalence of female genital mutilation/cutting in African countries. Campbell systematic reviews. 2012;8(1):1-55.

130. Ricardo C, Eads M, Barker G. Engaging Boys and Young Men in the Prevention of Sexual Violence: A systematic and global review of evaluated interventions . 2011; Available from: https://reliefweb.int/sites/reliefweb.int/files/resources/menandboys.pdf

131. Ruiz-Casares M, Lilley S, Thombs BD, Platt RW, Scott S, Isdijoso W. Protocol for a cluster randomised controlled trial evaluating a parenting with home visitation programme to prevent physical and emotional abuse of children in Indonesia: the Families First Programme. PUB Med [Internet]. 2019 Jan;9(1):e021751. Available from: https://pubmed.ncbi.nlm.nih.gov/30782674/

132. Sawasdipanich N, Srisuphan W, Yenbut J, Tiansawad S, Humphreys JC. Effects of a cognitive adjustment program for Thai parents. Wiley Online Libr [Internet]. 2010 Jun; Available from: https://onlinelibrary.wiley.com/doi/abs/10.1111/j.1442-2018.2010.00531.x

133. Schultz P. School subsidies for the poor: evaluating the Mexican Progresa poverty program. Sci Direct [Internet]. 2004 Jun; Available from: https://www.sciencedirect.com/science/article/abs/pii/S0304387803001858

134. Sedalacek G, Orazem P. Limiting Child Labor Through Behavior-Based Income Transfers: An Experimental Evaluation of the PETI Program in Rural Brazil. Res Gate [Internet]. 2002 Jan; Available from: https://www.researchgate.net/publication/242757079_Limiting_Child_Labor_Through_Behavior-Based_Income_Transfers_An_Experimental_Evaluation_of_the_PETI_Program_in_Rural_Brazil

135. Shereen U, Esca S, Susan G, Garth J. Achieving social change on gender-based violence: a report on the impact evaluation of Soul City’s fourth series. Soc Sci Med [Internet]. 2005 Dec;61(11):2434–45. Available from: https://www.ncbi.nlm.nih.gov/pubmed/16006028

136. Siaplay M. The impact of social cash transfers on young adults’ labor force participation, schooling, and sexual behaviors in South Africa. PMC [Internet]. 2011 Jun;33(5):675–696. Available from: https://www.ncbi.nlm.nih.gov/pmc/articles/PMC5951115/

137. Skeen S, Tomlinson M. A public health approach to preventing child abuse in low‐ and middle‐income countries: A call for action. 2013; Available from: https://onlinelibrary.wiley.com/doi/abs/10.1080/00207594.2012.737467

138. Santini PM, Williams LC. Parenting programs to prevent corporal punishment: A systematic review. Paidéia (Ribeirão Preto). 2016 Apr;26(63):121-9.

139. Ssenyonga J, Hermenau K, Nkuba M, Hecker T. Reducing violence against children by implementing the preventative intervention Interaction Competencies with Children for Teachers (ICC-T): study protocol for a cluster randomized controlled trial in Southwestern Uganda. 2018; Available from: https://trialsjournal.biomedcentral.com/articles/10.1186/s13063-018-2827-9

140. Sudhanshu H, Peterman A, Huang C, Halpern C, Pettifor A, Thirumurthy H. Impact of the Kenya Cash Transfer for Orphans and Vulnerable Children on early pregnancy and marriage of adolescent girls. Pub Med [Internet]. 2015 Sep;36–45. Available from: https://www.ncbi.nlm.nih.gov/pubmed/26246032

141. UNICEF. Tanzania Youth Study of the Productive Social Safety Net (PSSN) Evaluation: Endline Report. [Internet]. 2018. Available from: https://www.unicef-irc.org/publications/942-.html

142. Upton S, Farice G, Furman R. Meta-analysis of evaluations on ILO child labour programmes and projects in Africa, 2009-2014. 2017;

143. Vandana C, Sujata S, Rajesh S. Review of Randomized Controlled Trials on Psychological Interventions in Child Sexual Abuse: Current Status and Emerging Needs in the Indian Context. Pubmed [Internet]. 2016 Aug;38(4):279–84. Available from: https://www.ncbi.nlm.nih.gov/pubmed/27570336

144. Verma RK, Julie P, MV S, Khandekar S, AK S, Das SS, et al. Promoting gender equity as a strategy to reduce HIV risk and gender-based violence among young men in India. USAID [Internet]. 2008; Available from: http://menengage.org/wp-content/uploads/2014/06/Promoting-Gender-Equity-as-a-Strategy.pdf

145. Vision W. Impact of multi-purpose cash assistance on child labour among refugees among Syrian refugee children in Bekaa, Lebanon [Internet]. 2018. Available from: https://www.wvi.org/sites/default/files/Impact of Multi-Purpose Cash Assistance on Child Labour among Syrian Refugee Children in Bekaa%2C Lebanon.pdf

146. Waigwa S, Doos L, Bradbury-Jones C, Taylor J. Effectiveness of health education as an intervention designed to prevent female genital mutilation/cutting (FGM/C): a systematic review. Pub Med [Internet]. 2018 Apr;15(1):62. Available from: https://pubmed.ncbi.nlm.nih.gov/29650025/

147. Weatherley R, ABS H, Noralina O, John M, Preusser N, Yong M. Evaluation of a school-based sexual abuse prevention curriculum in Malaysia. Sci Direct [Internet]. 2012 Jan; Available from: https://www.sciencedirect.com/science/article/pii/S0190740911003446

148. Wessells M. What are we learning about protecting children in the community? An inter-agency review of evidence on community-based child protection mechanisms. Executive summary. UNICEF [Internet]. 2009 Nov; Available from: https://www.unicef.org/wcaro/What_We_Are_Learning_About_Protecting_Children_in_the_Community_Full_Report.pdf

149. Knerr W, Gardner F, Cluver L. Parenting and the prevention of child maltreatment in low-and middle-income countries: a systematic review of interventions and a discussion of prevention of the risks of future violent behaviour among boys. Pretoria: Sexual Violence Research Initiative, Medical Research Council, and the Oak Foundation. 2011.

150. Del Carpio XV, Macours K. The Impact of Conditional Cash Transfers on Child Labor Allocation in Nicaragua | The Abdul Latif Jameel Poverty Action Lab [Internet]. 2006. Available from: https://www.povertyactionlab.org/evaluation/impact-conditional-cash-transfers-child-labor-allocation-nicaragua

151. Zhang Y, Lochocki L, Meza-Cordero J. Child labour elimination actions for real change (CLEAR) project: Final report. 2016 Mar; Available from: https://www.impaqint.com/sites/default/files/files/CLEAR_FinalEvaluationReport_IMPAQ_toECLT%5B1%5D.pdf

152. Zwi K, Woolfenden S, Wheeler D, O’Brien T, Tait P, Williams K. School-based education programmes for the prevention of child sexual abuse. Campbell Collab [Internet]. 2015;11. Available from: <http://www.epistemonikos.org/documents/302b8a94ada060e49a62b74ff5498e45bc59e290>

**Online supplements**

***List of online supplements (online visualisation snapshots)***

1. ***Rows as interventions and columns as region***


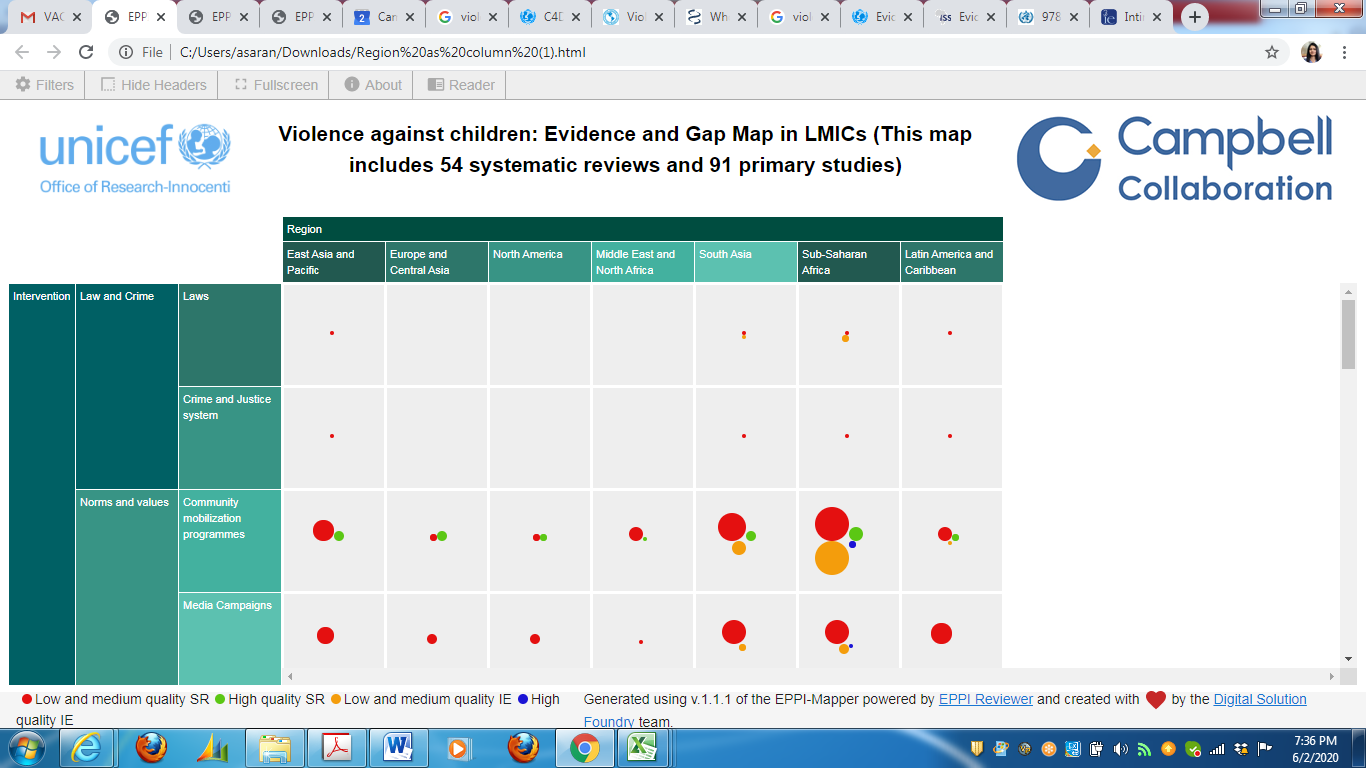


1. Rows as interventions and columns as additional filters (forms of violence and perpetrators)


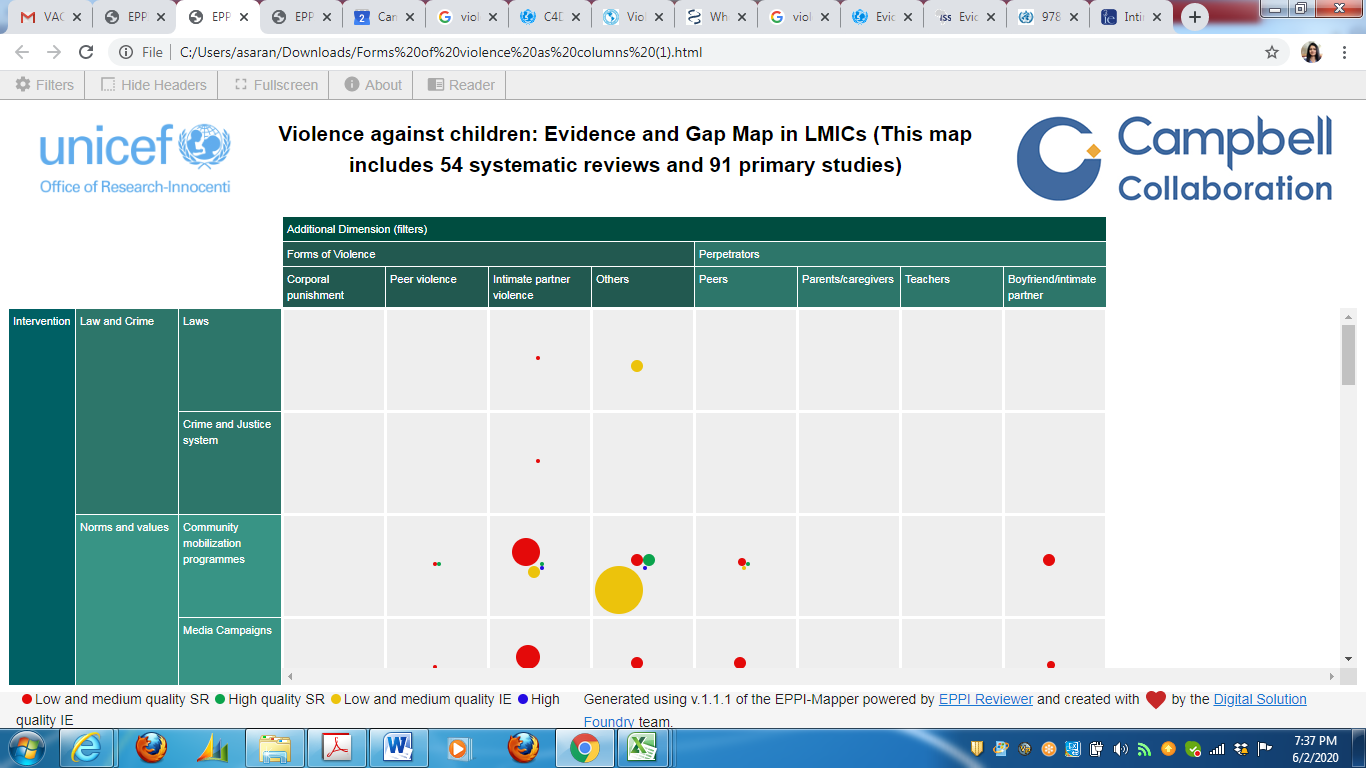


1. Rows as interventions and columns as outcomes


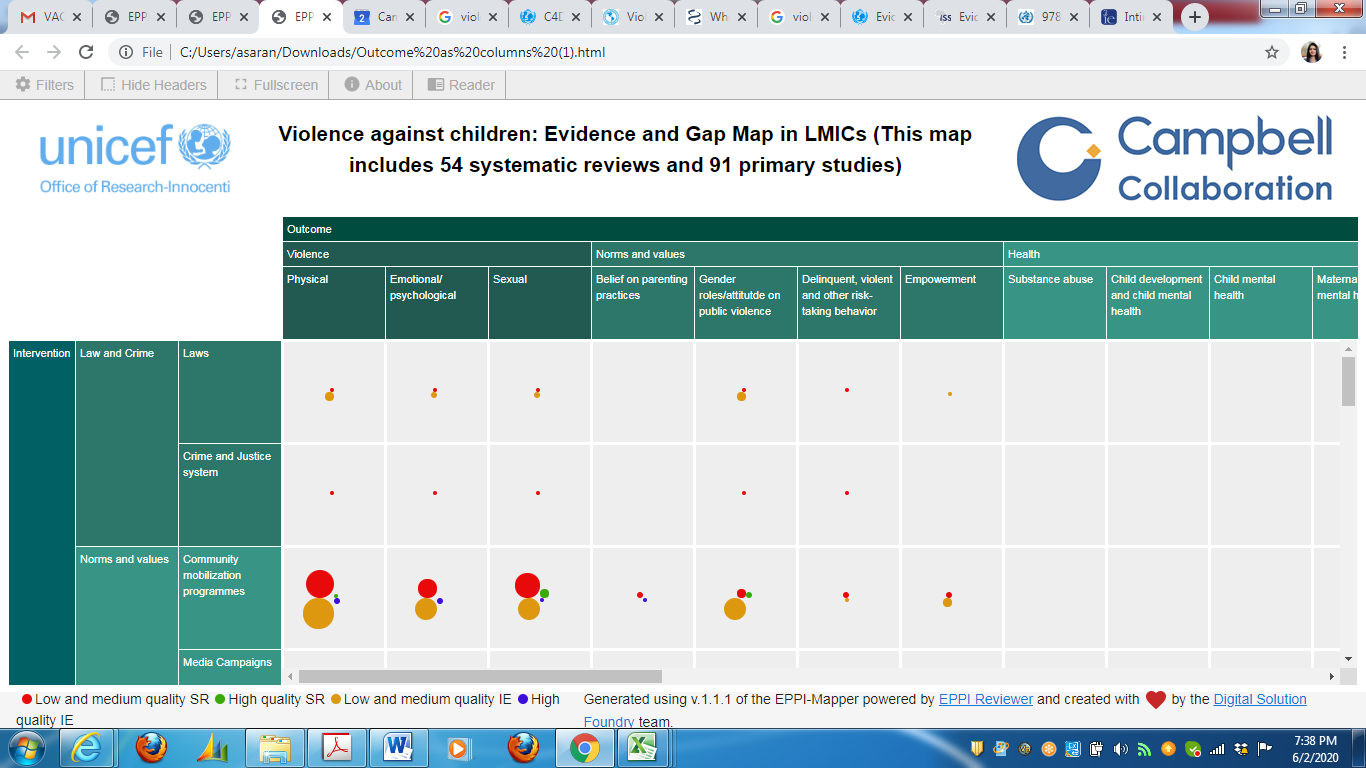

Supplement: Supplementary file 1 — Supporting information [file CL2-16-e1120-s001.docx]
